# Including state-of-the-art physical understanding of thermal vacancies in Calphad models

**DOI:** 10.1038/s41598-022-16926-5

**Published:** 2022-08-04

**Authors:** A. Obaied, I. Roslyakova, M. To Baben

**Affiliations:** 1 ICAMS, Ruhr-University Bochum, Universitaetstr. 150, 44801 Bochum, Germany; 2 GTT-Technologies, Herzogenrath, Germany

**Keywords:** Materials science, Physics

## Abstract

A physically sound thermochemical model accounting for explicit thermal vacancies in elements and alloys is presented. The model transfers the latest theoretical understanding of vacancy formation into the Calphad formalism where it can extend currently available thermodynamic databases to cover vacancy concentrations without a complete re-assessment. The parametrization of the model is based on ab initio-calculated enthalpy of vacancy formation and two model parameters describing the excess heat capacity of vacancy formation. Excellent agreement is obtained with temperature-dependent vacancy concentrations and elemental heat capacities while reasonable extrapolation of phase stability to high temperatures is ensured. Extrapolation to multicomponent systems is reasonable and the long-standing Neumann–Kopp related problem in the Calphad community is solved since multicomponent solid solutions will no longer show fingerprints of elemental heat capacity peaks at their melting points. FCC-Ag, FCC-Al and FCC-Cu, FCC-Zn, FCC-Ni, BCC-Ti, and BCC-W are used as a demonstration, along with the Cu–Zn binary system.

## Introduction

Thermal vacancies are an important structural defect in metals as their effects are strongly seen in materials properties such as diffusion^1^ and precipitation processes^2^. For example, the role of vacancies in natural aging of aluminium alloys^3,4^ and thermal stability of hard coatings^5^ have only recently been understood. They also affect thermophysical quantities, such as heat capacity and thermal conductivity, close to the melting point^6^. They are considered a special thermodynamic component, as it is impossible to control their external chemical potential or consider them in a materials balance^7^.Therefore, it would be important to account for vacancy contributions in the Gibbs energy while modelling materials properties^8^. However, this is only rarely done in the Calphad formalism, the most successful thermodynamic modelling formalism describing inorganic materials.

The Calphad approach has been a stable entity in materials research since the 1970s due to its success in thermodynamically modelling multicomponent systems. This phenomenological approach uses thermochemical and phase stability data to model the Gibbs energy of each phase^9–11^. Despite the important effect that vacancies can have on multicomponent systems, there is no commonly accepted approach to account for explicit thermal vacancies contributions in Calphad-type assessments^8^. In recent years, 3rd generation Calphad databases have been introduced to develop more physically-based models that can extend the thermodynamic description of materials over the whole temperature range^12–17^. However, thermal vacancies effects are still not included in a physically-consistent way^7,8,18,19^.

On the contrary, it became recently common to accept unphysical values to thermodynamic quantities, such as a non-zero Gibbs energy of a hypothetical crystal made up of vacancies, i.e. “nothing” non-zero enthalpy or has negative entropy^5,7,18–22^. In some parametrizations, even negative entropy of vacancy formation at low temperature can be observed^21^. Additionally, The Calphad method’s main principle is modelling “from bottom to top”^10^, which means that a reliable extrapolation to any multicomponent system will not only depend on the unary system descriptions and how compatible they are at higher temperatures, but also on the extrapolation method, which is usually the Neumann–Kopp rule^23^. While this method has proven effective in several cases, sometimes it can yield inconsistent results and be even misleading^23–25^.

The physical basis on which a Calphad model must be developed is well understood. Thermal vacancies have been investigated extensively both theoretically^26–29^ and experimentally since the 1950’s^6^. An extensive review of vacancies in pure metals was put forward by Kraftmakher^6^. He concluded that “defect contributions to the specific heat of metals are much larger than nonlinear effects of the anharmonicity and can be separated without crucial errors”. This implies that it is possible and necessary to separate these different contributions in the Calphad model. Recently, several first-principles calculations^27,30–32^ have expanded the understanding of vacancy formation on an atomic scale in two important directions: First, these studies challenged the previous consensus that the entropy of vacancy formation was temperature-independent^33^, which was based on the Arrhenius approximation, and showed that it is linearly dependent on temperature^33^. Second, different contributions to heat capacity have been quantified for several elements. While the required computational efforts prohibit the calculation of these contributions for multicomponent systems, for all elements studied high-temperature heat capacity changes (in first-order approximation) linearly with temperature if vacancies are ignored^30,33–35^. Therefore, a Calphad model should be based on a temperature-dependent entropy of vacancy formation, and it is reasonable to assume that the heat capacity of the hypothetical vacancy-free metal changes linearly with temperature.

In this work, we combine the latest understanding of thermal vacancy formation from experimental and theoretical points of view into a thermochemical model that can be applied to and easily parametrized for multicomponent solid solutions up to very high temperatures. The thermochemical model is based on the compound energy formalism (CEF) and is fully compatible with existing CALPHAD databases but removes unphysical assignment of non-zero Gibbs energy of a hypothetical crystal consisting only of vacancies. It is also consistent with the recent conclusion that excess entropy of vacancy formation is temperature-dependent and zero at 0 K. The parametrization is reduced to two parameters, where one is accessible by computationally cheap calculations based on density functional theory and the other is fitted to experimentally determined vacancy concentrations. At the same time, it is ensured that the model extrapolates well to temperatures up to 6000 K, making it generally applicable to all solids. As a side effect, the long-standing Neumann–Kopp related problem in the CALPHAD community is solved so that multicomponent solid solutions will no longer show fingerprints of elemental heat capacity peaks at their respective melting points. As study systems, FCC-Ag, FCC-Al and FCC-Cu, FCC-Ni, BCC-Ti, and BCC-W are used. The model behavior for solid solutions is demonstrated for the FCC phase in the Cu–Zn system.

Due to its effects on materials properties, the formation of vacancies has been experimentally investigated for decades. Kraftmakher has compiled an excellent review of the known literature from an experimental point of view^6^. In pure metals, vacancies form because of the configurational entropy, while bond breaking leads to a positive vacancy formation enthalpy. In heat capacity measurements of pure metals, a non-linear increase in heat capacity is observed because with increasing temperature the vacancy concentration increases^6^. For a pure metal, Gibbs energy of vacancy formation ${G}^{f}(T)$ is considered the main quantity to describe vacancies and relates to the equilibrium vacancy concentration^7^, ${c}_{Va}^{eqm}$, by Eq. (1):

1 $$ c_{Va}^{eqm} = \exp \left( { - G^{f} \left( T \right)/k_{B} T} \right) $$

Here, ${k}_{B}$ is the Boltzmann constant and $T$ temperature. Calorimetric measurements are often relied upon to obtain ${c}_{Va}^{eqm}$ values^6^. Despite the considerable impact vacancies have on mechanical and thermophysical properties, it is still rather difficult to accurately obtain accurate measurements of vacancy concentrations along the whole temperature range. Furthermore, experimentally measuring vacancy concentrations is a limited process that demands a complicated combination of different techniques^33^. The differential dilatometry (DD) method is considered the most accurate technique for measuring vacancy concentrations following the standards set by Hehenkamp^36^. Still, the results are most reliable between the melting point and about 80% of ${T}_{melt}$ only, as the results obtained below this point are too scattered to be reliable^6,33^. Equation (1) can easily be derived from a Gibbs energy minimization argument for a system consisting of one metal and vacancies, but it does not apply to solid solutions of metals or compounds such as $\left( {{\text{Ti}},{\text{Al}},{\text{Va}}} \right)\left( {{\text{N}},{\text{Va}}} \right)$^5^ and is completely inappropriate for phases such as $\left(\mathrm{Ti},\mathrm{Va}\right)\left(\mathrm{O},\mathrm{Va}\right)$ and $\left(\mathrm{V},\mathrm{Va}\right)\left(\mathrm{O},\mathrm{Va}\right)$ that exhibit vacancy concentrations on both metal and oxygen sublattice in the order of 20%^37^.

**Figure 1:**
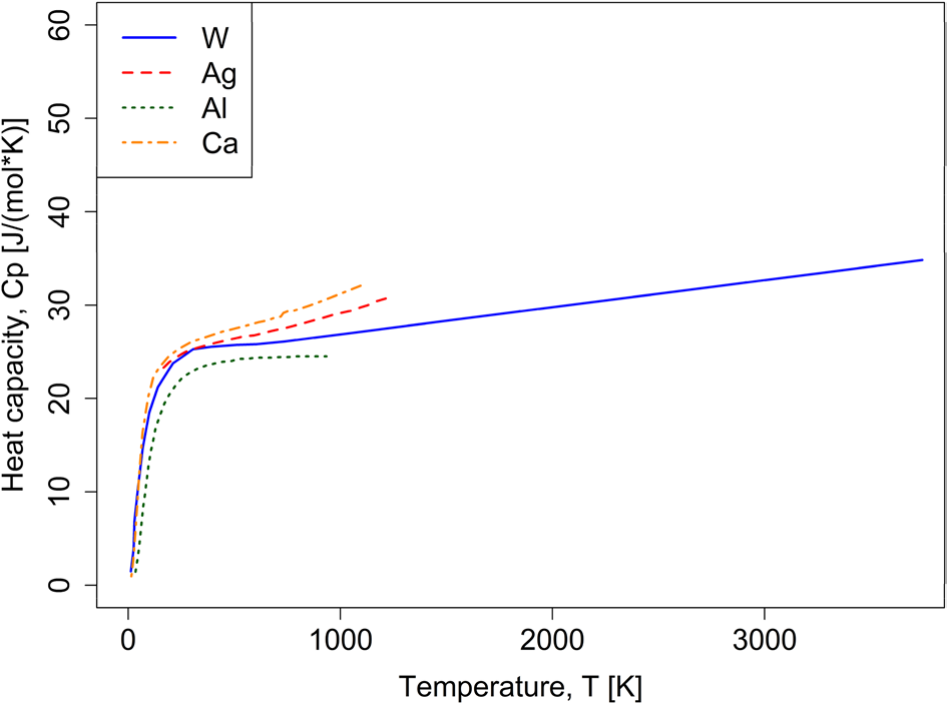
DFT-based heat capacity calculations without vacancy contributions [quasiharmonic, electronic and anharmonic contributions only] for W^34^, Al^34^, Ca^35^ and Ag^30^. Each line ends at the melting point of its respective element.

One of the downsides of experimental calorimetric measurements is that it is impossible to control vacancy concentrations. On the other hand, recent computational advances have enabled atomistic simulations that have significantly enhanced our understanding of vacancy thermodynamics^6^. Most importantly, Glensk et al.^38^ and Gong et al.^31^ have shown that the entropy of vacancy formation is temperature-dependent and reduces to zero at 0 K. This indicates that the Gibbs energy of vacancy formation must be described by a combination of enthalpy of vacancy formation and heat capacity of vacancy formation. Additionally, several ab-initio based studies have disentangled heat capacity contributions for vacancy-free metals^30,33–35^. As is shown in Fig. 1, heat capacity of the vacancy-free metal can be approximated well by a linear temperature-dependence at high Temperatures (~ above 2/3 of the melting point)^30,33–35^.

The CEF has been implemented on a wide scale in the Calphad method to describe thermodynamic properties of crystalline phases and to determine their phase equilibria^10,39^. With suitable parametrization, the CEF would be applicable also for solid solutions containing vacancies. In the last decades, several attempts have been made to model vacancies within the Calphad approach using the CEF, where it was necessary to assign a value for the Gibbs energy of a hypothetical pure vacancy solution endmember. While there is little debate that the Gibbs energy of pure vacancy (= nothing) should be equal to zero^7,19,40^, out of practical considerations Gibbs energy of pure vacancies is often described as non-zero, 60 kJ/mol^7^, 0.2 RT^17,20,21^, 10 RT^18^ to 30 T^22^. The reason has been described by Franke^20^ who provided a detailed mathematical analysis of the Gibbs energy of the solid solution for different parametrization, showing that an artificial local minimum in the Gibbs energy of the solution is generated for vacancy concentrations close to 100% due to the configurational entropy contribution if the Gibbs energy of the pure vacancy endmember is set to zero^20^. This artificial Gibbs energy minimum can be avoided if the pure vacancy endmember is sufficiently de-stabilized by assigning a positive Gibbs energy^20^. Another, rarely applied solution, is to limit the range of applicability of the solid solution description by setting a maximum vacancy concentration, e.g. 40%^22^. We will show below that both options -setting Gibbs energy of pure vacancies to a small but positive value or setting it to zero and define a range of applicability- can be used and result in equivalent results for vacancy concentrations of interest.

## Model description

In the CEF, Gibbs energy of an n-component solution containing vacancies is given by

2 $$ G\left( {y_{Va} ,y_{1} , \ldots y_{n} } \right) = \left[ {\begin{array}{*{20}c} {\mathop \sum \limits_{i = 1}^{n} y_{i} G_{i} + RT\mathop \sum \limits_{i = 1}^{n} y_{i} \ln y_{i} + y_{Va} G_{Va} + RTy_{Va} \ln y_{Va} + \mathop \sum \limits_{i = 0} \mathop \sum \limits_{j = i} y_{i} y_{j} \left( {y_{i} - y_{j} } \right)L_{E}^{i,j} + } \\ {y_{Va} \mathop \sum \limits_{i = 1}^{n} y_{i} L_{E}^{i,Va} + PV} \\ \end{array} } \right], $$

where ${y}_{Va}$ denotes the vacancy mole fraction, ${G}_{Va}$ is the Gibbs energy of a hypothetical pure-vacancies end-member, that is an empty crystal lattice occupied by vacancies only,${y}_{i}$ and ${G}_{i}$ are the mole fraction and the Gibbs energy of the defect-free pure elements $\left(1,\dots n\right)$ in the solution, respectively. $R$ is the universal gas constant. ${L}_{E}^{i,j}$ denotes the excess energy interaction parameter between two atoms *i* and *j* and ${L}_{E}^{i,Va}$ between a host atom *i* and vacancy $Va$, both of which can be extended using Redlich–Kister (R–K) polynomials^41^. $P$ denotes pressure and $V$ represent the volume of the solution.

For simplicity, a pure metal A is considered here. The CEF extends the Gibbs energy of the solid solution between two end-members, a defect-free $A$ crystal and a hypothetical vacancy-filled crystal^42^. For one mole of element $\mathrm{A}$, the Gibbs energy is

3 $$ G = y_{A} G_{A} + y_{Va} G_{Va} + RTy_{A} \ln y_{A} + RTy_{Va} \ln y_{Va} + y_{A} y_{Va} L_{E}^{A,Va} + PV, $$

${y}_{A}$ and ${G}_{A}$ are the mole fraction and the Gibbs energy of the defect-free crystal of $A$, respectively. The excess Gibbs energy parameter ${L}_{E}^{A,Va}$ is

4 $$ L_{E}^{A,Va} = \Delta G^{e} \left( T \right) = \Delta H^{e} \left( T \right) - T\Delta S^{e} \left( T \right), $$

where $\Delta {H}^{e}\left(T\right)$ is the excess enthalpy and $\Delta {S}^{e}\left(T\right)$ is the excess entropy. Note that both are assumed to be temperature-dependent.

Guan and Liu argued that it is necessary to consider the effect of pressure in the Gibbs energy model, to account for its impact on the total volume of the system, by including the $PV$ term in a substitutional solution model that includes a vacancy^19^. Therefore, $V$ is defined using the following formula:

5 $$ V = y_{A} V_{A} + y_{Va} V_{Va} + y_{A} y_{Va} L_{V}^{A,Va} , $$

where ${L}_{V}^{A,Va}$ is the excess volume interaction parameter between the host atom $A$ and vacancy $Va$, extended using Redlich–Kister (R–K) polynomials^41^. Since we focus on Gibbs energy of vacancy containing metals and the $PV$ term is small compared to the enthalpy of vacancy formation, we ignore second-order effects such as volume expansion and compressibility, for which more sophisticated models could however be used.

As introduced above, the choice for ${G}_{Va}$ has been under heavy debate. In many publications (e.g. Oates et al.^7^, Abe et al.^18^, Guan and Liu^19^, and Rogal et al.^8^, and) it is pointed out that a “crystal” without nuclei or electrons should collapse to zero volume for any pressure (> 0) and will contribute zero Gibbs energy and zero volume to a system. In that case, all thermodynamic properties of vacancy-containing crystals are described using the excess parameters (${L}_{E}^{A,Va},{L}_{V}^{A,Va}$). This is reasonable since the excess parameters describe the interaction between the endmembers $A$ and $Va$, while the endmember’s Gibbs energies describe the $A$-$A$ and $Va$-$Va$ interactions. Due to the small vacancy concentrations observed experimentally, the Gibbs energy of monovacancy formation should not be described by $Va$-$Va$ interactions but by $A$-$Va$ interactions. Divacancies are outside the scope of the current paper.

To tackle the mathematical problem presented by Franke^20^, i.e. the local minimum of the Gibbs energy for a vacancy concentration close to 100%, we consider both known options: First, we limit the range of applicability of the Gibbs energy model by the condition ${y}_{Va}<0.2$, similar to a suggestion by Dinsdale et al.^22^. This limit is chosen to ensure that the very large vacancy concentrations observed experimentally, e.g. in Titanium and Vanadium monoxides in the range of ^~^20% on both sublattices of the Halite structure, are being covered. Note that it is only necessary to apply this limit for one sublattice (e.g. the metal sublattice of the Halite structure), while the vacancy concentration on the other sublattice (e.g. the oxygen sublattice of the Halite structure) may remain unconstrained (which enables the continuous description from Halite to FCC metal with metal vacancies and interstitially dissolved oxygen). Since most Calphad software packages do not offer such an option, we compare this to an arbitrarily chosen value of ${G}_{Va}=2\frac{J}{K mol}T$. This contributes an unphysical entropy of vacancy formation at 0 K of $-2\frac{J}{K mol}$ which is compensated for by the excess Gibbs energy parameter ${L}_{E}^{A,Va}$ which is still the most important parameter describing vacancy formation. This is possible since a crystal at 0 K exhibits an equilibrium vacancy concentration of zero which is perfectly in line with the ideal dilute limit.

Both parametrizations satisfy the condition that approaching the pure vacancy endmember from two different host crystals must result in the same lattice stability^43^ and as we show in the supplementary Fig. S1 lead to equivalent descriptions for all vacancy concentrations of interest. All figures in the main part of this paper are identical for the two different model approaches.

Calphad databases rely on experimentally obtained heat capacity measurements to assess unary and higher-order systems. Since the effects of vacancy concentration cannot be suppressed during an experiment, the large enthalpy of formation of vacancies leads to a noticeable non-linear contribution in experimentally obtained heat capacity data. The ${G}_{i}$ term of Eq. (2) must however describe the defect-free crystal. This concern was raised by Abe et al.^18^, where the effects of vacancies on phase equilibria were accounted for twice. It was argued to be small and therefore ignored. However, it is clear that this is not the case for all elements^6^. Here, the implicit thermal vacancy effects are removed by adjusting the pure Gibbs energy description for the pure metal end members. According to Kraftmakher^6^, the thermal vacancies effects are limited under a certain temperature cutoff limit, usually $2/3{T}_{melt}$, where the their effects can be ignored. This is consistent with state-of-the-art atomistic simulations of high-temperature heat capacity of elements distinguishing harmonic, electronic and anharmonic contributions to heat capacity that indicate a rather linear high-temperature behavior, see e.g. W^34^, Al^34^, Ca^35^, and Ag^30^ in Fig. 1. Therefore, the existing Gibbs energy descriptions^44^ above ${T}_{cutoff}=2/3{T}_{melt}$ are modified to a linear extrapolation to exclude the implicit thermal vacancies effects.

**Figure 2:**
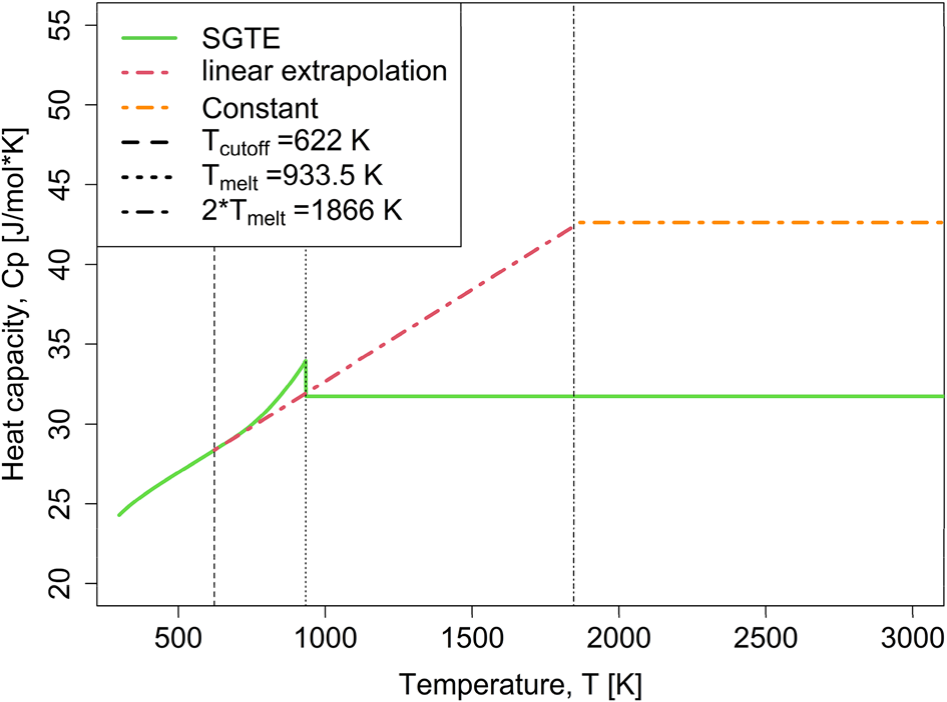
Modified heat capacity description for FCC-Al used for applying the proposed model (dotted-point line) compared with SGTE^44^ description (solid line).

In Calphad assessments, a constant value of the stable solid heat capacity description is usually adopted after ${T}_{melt}$^10^. This avoids artificial re-stabilization of solid phases at very high temperatures due to too large heat capacities above the melting point^24,25,45^. Additionally, it is common in Calphad assessments to linearly interpolate heat capacity of multicomponent phases between the elements (“Neumann–Kopp rule”). This usually causes unrealistic heat capacity peaks for multicomponent phases at the melting points of the constituting elements^24,46^. To avoid the fingerprint from the elemental melting points in alloys’ heat capacities we extend the linear extrapolation of elemental heat capacity beyond the melting point, however -to avoid re-stabilization at very high temperatures– only up to $2{*T}_{melt}$, above which a constant value is adopted. Figure 2 shows the adopted heat capacity description for FCC-Al as example. The underestimation of heat capacity at the melting point is not a concern since at this temperature vacancies contribute to the experimentally measured heat capacity and will be described explicitly.

**Figure 3:**
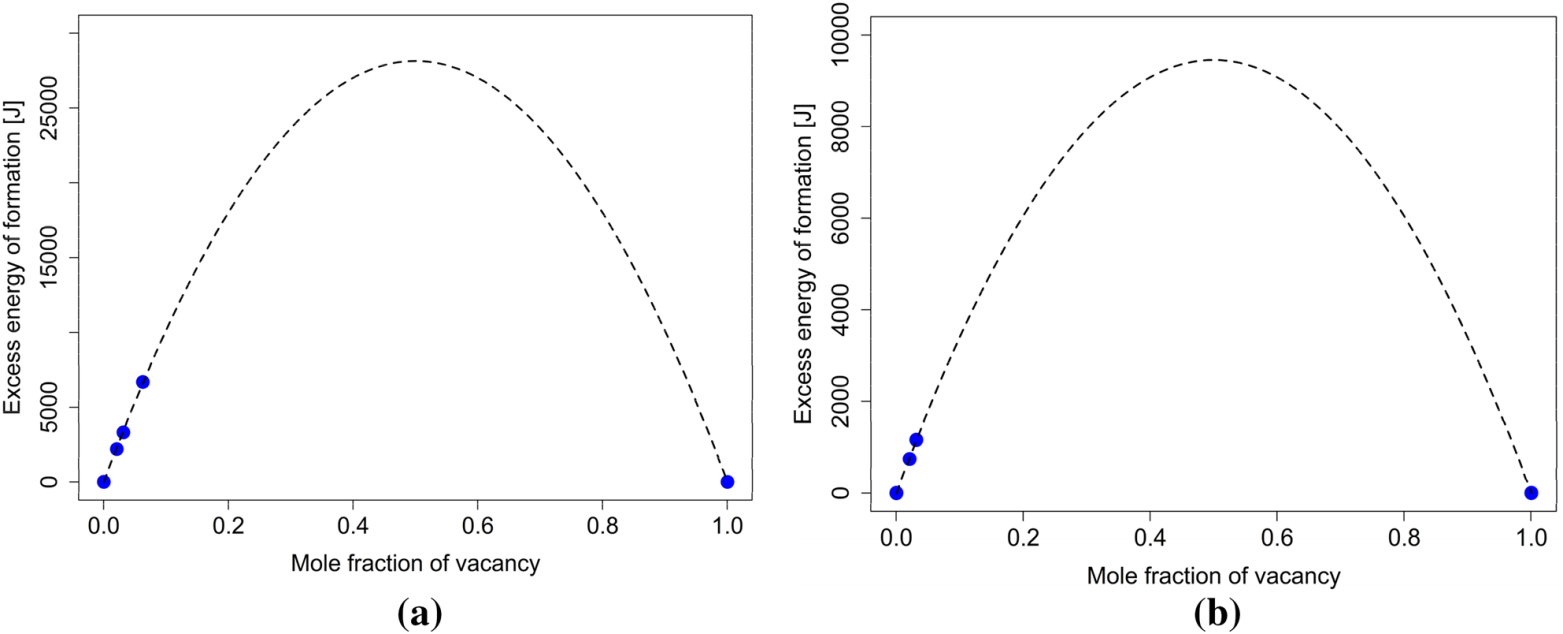
Excess energy of formation at 0 K per one mole of lattice sites (**a**) for FCC-Cu and (**b**) for FCC-Zn.

As stated above, in this work, the thermal vacancies contributions to the solution’s properties are mainly accounted for using the interaction parameters for excess energy ${L}_{E}^{A,Va}$ and excess volume ${L}_{V}^{A,Va}$. Evaluating the interaction parameter ${L}_{E}^{A,Va}$ requires determining the corresponding $\Delta {H}^{e}\left(T\right)$ and $\Delta {S}^{e}\left(T\right)$ quantities. $\Delta {H}^{e}\left(T\right)$ can be expressed as

6 $$ \Delta H^{e} \left( T \right) = \Delta H^{e} \left( {0 {\text{K}}} \right) + \mathop \smallint \limits_{0K}^{T} \Delta C_{P}^{e} \left( T \right) dT, $$

where $\Delta H^{e} \left( {0 {\text{K}}} \right)$ is the excess enthalpy of formation at 0 K, and this quantity can be easily evaluated using ab initio calculations. $\Delta C_{P}^{e} \left( T \right)$ is the excess heat capacity of vacancy formation. The estimation of $\Delta H^{e} \left( {0 {\text{K}}} \right)$ quantity was carried out using multiple ab initio calculations for each investigated unary system (FCC-Al, FCC-Ag, FCC-Ni, BCC-W, FCC-Cu, FCC-Zn). The results were fitted using a simple quadratic function, yielding the value of $\Delta H^{e} \left( {0 {\text{K}}} \right)$. This can be seen for FCC-Cu and FCC-Zn in Fig. 3. As can be seen here, all the obtained data points (up to 6.25% of vacancies) can be well described using a quadratic fit. Additionally, a comparison between the calculated $\Delta H^{e} \left( {0 {\text{K}}} \right)$ values from this work and corresponding experimentally determined values are shown in Table S1. The calculated values are in reasonable agreement with experimental ones.

$\Delta {S}^{e}\left(T\right)$ is then defined as:

7 $$ \Delta S^{e} \left( T \right) = \Delta S^{e} \left( {0 {\text{K}}} \right) - \mathop \smallint \limits_{0K}^{T} \frac{{\Delta C_{P}^{e} \left( T \right)}}{T} dT, $$

where $\Delta S^{e} \left( {0 {\text{K}}} \right)$ is the excess entropy of formation at 0 K and is equal to zero^31,38^ in case of ${G}_{Va}=0$ and to $2\frac{J}{K mol}$ in the case of ${G}_{Va}=2\frac{J}{K mol}T$ which also leads to zero entropy of vacancy formation at 0K^31,38^. It was often assumed that vibrational entropy of defect formation was small and therefore ignorable^47^, which lines up with an Arrhenius-like description that presumes a constant entropy and enthalpy of formation. However, non-Arrhenius diffusion behavior was consistently observed in later experiments for several pure elements, such as Na^48^, K^49^, Ag^50^, and V^51^, and it was then determined that the Arrhenius approximation needed to be replaced by the Local Grüneisen Theory (LGT), which states that the temperature dependence of formation entropy can be approximated linearly for monovacancies and divacancies, which implies a temperature-dependent heat capacity of vacancy formation and scales in the Gibbs energy of vacancy formation by a ${T}^{2}$ approximation^33,38^. As will be demonstrated below, such a model extrapolates poorly to very high temperatures, so the following ${\Delta C}_{P}^{e}\left(T\right)$ expression was adopted:

8 $$ \Delta C_{P}^{e} \left( T \right) = - 2aT - 6bT^{2} , $$

where $a$ and $b$ are fitting parameters. Setting *b* = *0* reduces the model to the LGT approximation but will result in constantly increasing entropy of vacancy formation and consequently when extrapolating the model to temperatures well above the melting point the solid phase will restabilize. In addition, it was recently shown by Grabowski et al.^26^ that anharmonic vibrations and vacancies are strongly coupled and it can be expected that this coupling would increase further as vacancy concentrations increase in a hypothetical crystal superheated significantly above the melting point. Therefore, it is reasonable to assume that there is a non-linear ${\Delta C}_{P}^{e}\left(T\right)$ contribution at high-temperature and expand the function even further up to the second order in temperature. The choice of Eq. (8), makes it possible to re-write the $\Delta {H}^{e}\left(T\right)$ and $\Delta {S}^{e}\left(T\right)$ formulas as:

9 $$ \Delta H^{e} \left( T \right) = \Delta H^{e} \left( {0 {\text{K}}} \right) - aT^{2} - 2bT^{3} , $$

10 $$ \Delta S^{e} \left( T \right) = \Delta S^{e} \left( {0 {\text{K}}} \right) - 2aT - 3bT^{2} , $$

which results in the following simple equation for the excess Gibbs energy interaction parameter ${L}_{E}^{A,Va}$

11 $$ L_{E}^{A,Va} = \Delta G^{e} \left( T \right) = \Delta H^{e} \left( {0 {\text{K}}} \right) - T\Delta S^{e} \left( {0 {\text{K}}} \right) + aT^{2} + bT^{3} $$

**Figure 4:**
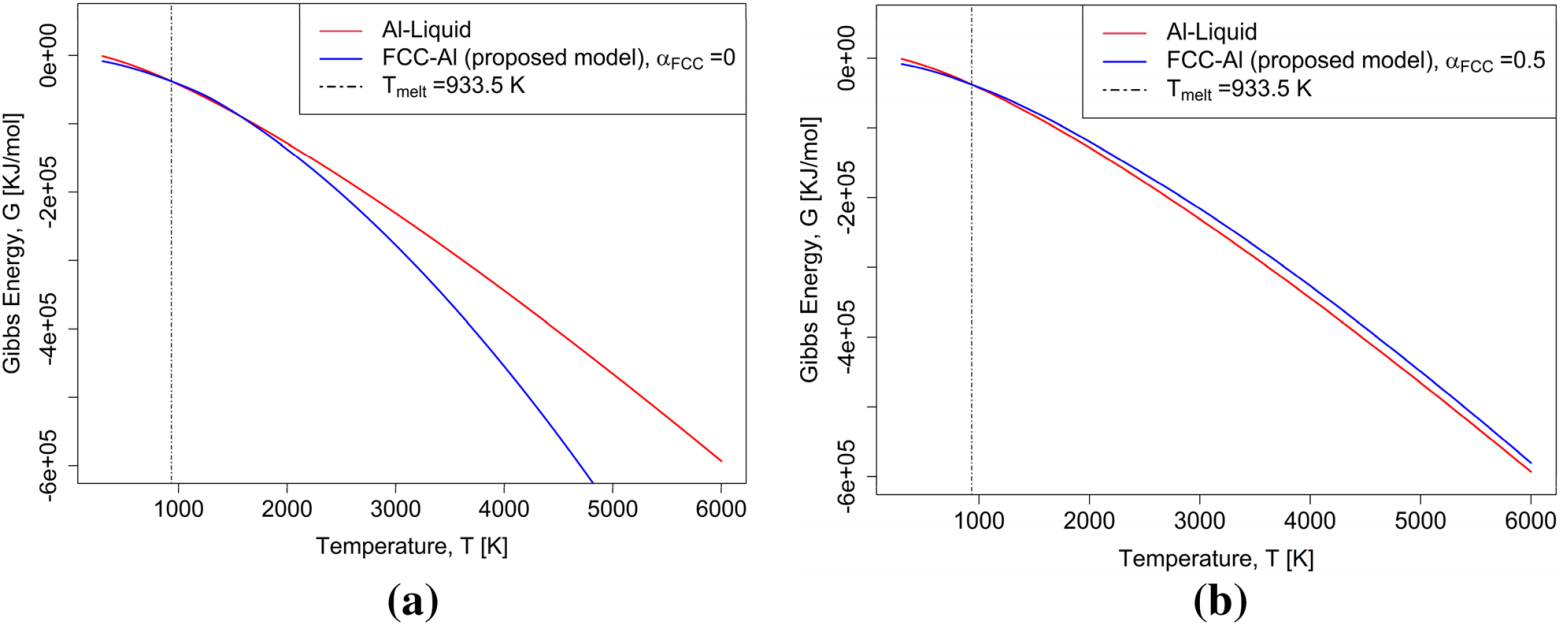
Gibbs energy description for FCC-Al using the proposed model compared with liquid phase^44^, (**a**) using $\alpha_{FCC}$  = 0 (**b**) $\alpha_{FCC}$  = 0.5.

It should be noted that at low temperatures, the $aT$ term dominates the entropy of vacancy formation while the $bT^{2}$ term is responsible for a well-behaving extrapolation to very high temperatures. The values of $a$ and $b$ are fitted to produce a final solution that satisfies two boundary conditions. (i) The first and hard condition is that the vacancy concentration at ${T}_{melt}$ matches the equilibrium vacancy concentration ${c}_{Va}^{eqm}$ at ${T}_{melt}$ measured experimentally. This is achieved by fitting the $a$ parameter in the first term of Eq. (8) with ${c}_{Va}^{eqm}$ at ${T}_{melt}$. (ii) The second and softer condition is that the resulting Gibbs energy description does not lead to re-stabilization at temperatures well above the liquidus temperature. This is achieved by determining the order of magnitude that the $b$ parameter in the second term of Eq. (8) is required to satisfy this condition. To reduce the degrees of freedom during fitting, we suggest coupling the two parameters $a$ and $b$. If b is set to zero unphysical re-stabilization of the solid phase happens at high temperatures, as shown in Fig. 4a applying the here-proposed methodology for FCC-Al while setting the value of the $b$ parameter to zero. FCC-Al then re-stabilizes at around 3000 K. A model ignoring this will not be applicable to hard coatings of (Ti, Al, Va)(N,Va)^5^, which is in Calphad databases modelled as the same phase as the FCC phase and where at least the TiN endmember is stable above 3000 K.

Assigning a constant value to $b$ applicable to all elements is not desirable since the $- 3bT^{2}$ term in Eq. (10) should play a role only at the melting temperature, not at temperatures significantly below that. Therefore, we connect the *b* term to the *a* term by a coupling parameter $\alpha$ and the melting temperature, ${T}_{melt}$ using the following equation:

12 $$ - 3b*T_{melt}^{2} = \alpha *2a*T_{melt} , $$

So that the entropy of vacancy formation at the melting point is given by:

13 $$ \Delta S^{e} \left( {T_{melt} } \right) = - 2aT_{melt} + \alpha *2a*T_{melt} = - 2aT_{melt} \left( {1 - \alpha } \right), $$

Thus, the $\alpha$ parameter is a measure of how much the $-3b{T}^{2}$ contribution in Eq. (10) reduces the $-2aT$ contribution to the entropy of vacancy concentration at the melting point. This procedure is in tradition of other works that have coupled Gibbs energy of vacancy formation parametrizations to the melting point^6^. As shown in Fig. 4a, for $\alpha$ = 0, re-stabilization of the solid phase occurs at high temperatures. For each element however, there is a lower limit of $\alpha$ that will ensure that the solid phase does not re-stabilize. Here, Al, Ag, Cu, Ni, Ti, and W have been studied to derive a universal coupling parameter $\alpha$ that ensures that artificial re-stabilization is avoided independent of the element. Using available equilibrium vacancy concentration ${c}_{Va}^{eqm}$ data from the literature^6^, the value of the $a$ parameter is fitted for different arbitrarily chosen values of $\alpha$ between 0 and 1 so that the value of the vacancy concentration at ${T}_{melt}$ would match the experimentally measured ${c}_{Va}^{eqm}$ value without re-stabilization. All elements show re-stabilization below 6000 K for $\alpha =0$ as shown in Fig. 4a. For all FCC-elements (Al, Ag, Cu, Ni) studied, the critical value of $\alpha$ is around 0.3, indicating that ${\alpha }_{FCC}=0.5$ can be used as recommended universal coupling parameter for all FCC elements studied here. The BCC elements studied here, Ti and W, require a larger value, which leads us to recommend the value of ${\alpha }_{BCC}$ = 0.75 adopted here. The reason for the larger value for Ti and W compared to the other elements is not clear and it is a task for future work to check whether this is a universally true for all BCC-metals. The interaction parameters for all assessed unary systems can be found in Table S2. Figure 4b shows that the Gibbs energy of FCC-Al extrapolates reasonable to 6000 K when the $b$ parameter is set using the coupling parameter ${\alpha }_{FCC}=0.5$. No re-stabilization is observed. It must be noted that the number of fitting parameters is reduced to one by the universal setting of $\alpha$. The single fitting parameter, $a$, is determined simply by the vacancy concentration at the melting point, ${c}_{Va}^{eqm}$. It is important to mention that the experimental ${c}_{Va}^{eqm}$ data used were obtained using the Differential dilatometry (DD) method, which is regarded as the most reliable method of measuring equilibrium vacancy concentrations^6^.

**Figure 5:**
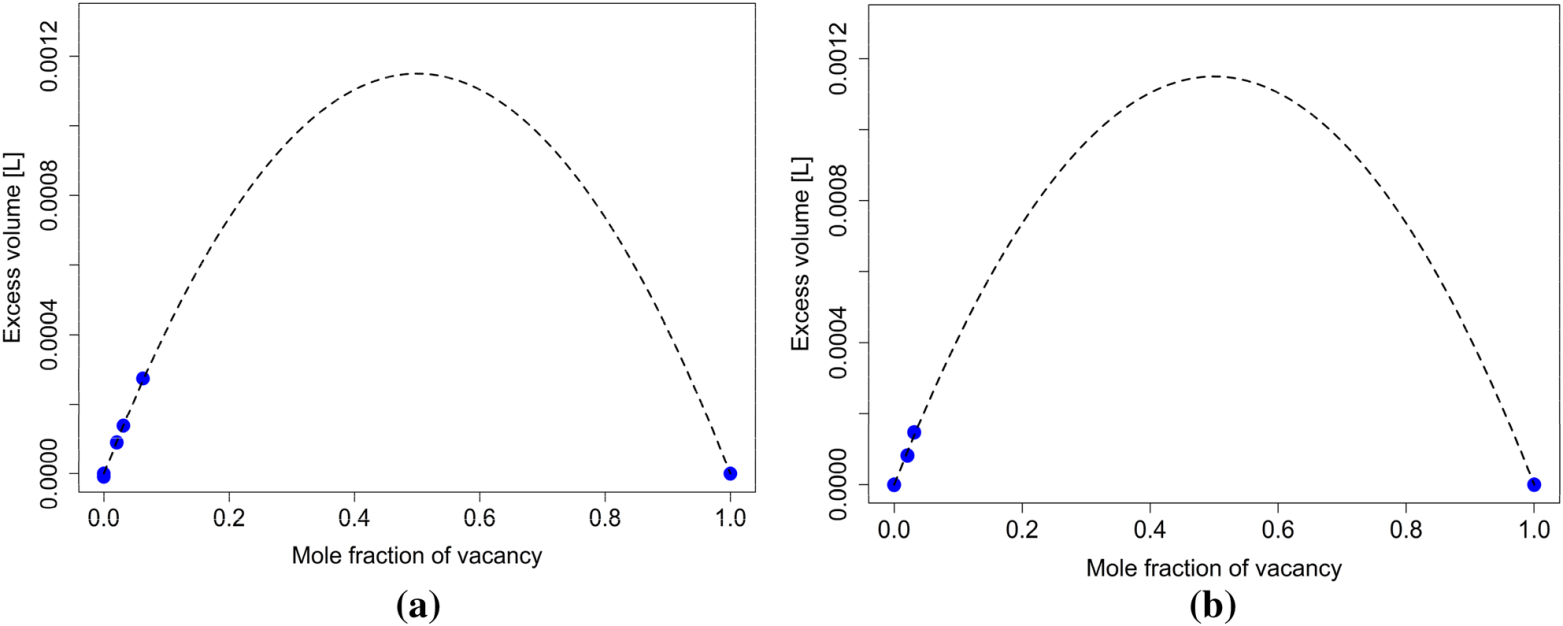
(**a**) Excess volume for FCC-Cu (**b**) excess volume for FCC-Zn.

The only parameter from Eqs. (2)–(13) not discussed extensively yet is the excess volume interaction parameter ${{\varvec{L}}}_{{\varvec{V}}}^{{\varvec{A}},{\varvec{V}}{\varvec{a}}}$. As mentioned earlier, the importance of accounting for the excess volume was explored previously by Guan and Liu^19^, who accounted for the significant impact that the vacancy formation has on the total volume of the system. We use a somewhat simpler approach by accounting for the effect of vacancy concentration on volume in the interaction parameter ${{\varvec{L}}}_{{\varvec{V}}}^{{\varvec{A}},{\varvec{V}}{\varvec{a}}}$. For this, ${{\varvec{V}}}_{{\varvec{m}}}$ was obtained from the same calculations used to determine $\Delta {\varvec{H}}^{{\varvec{e}}} \left( {{\mathbf{0}}\,{\mathbf{K}}} \right)$. The excess volume is shown in Fig. 5 for FCC-Cu and FCC-Zn. In parameter studies we observed that the values obtained for ${{\varvec{V}}}_{{\varvec{m}}}$ contributed very little to the overall Gibbs energies of the examined systems and could therefore be ignored.

In the following paragraph, the model behavior is analysed and benchmarked against literature data.

## Results and discussion

Here, the model performance is demonstrated with respect to heat capacity, vacancy concentration, Gibbs energy and entropy of vacancy formation, and the phase stability extrapolation to very high temperatures. Experimental data available in the literature that has not been used in the parametrization is shown.

### Comparing with experimental $C_{P}$ data

For all the investigated unary systems, a comprehensive literature survey was carried out to collect as many heat capacity datasets as possible. These datasets will provide a visual guideline to ensure that the proposed description does not account for the thermal vacancies twice.

**Figure 6:**
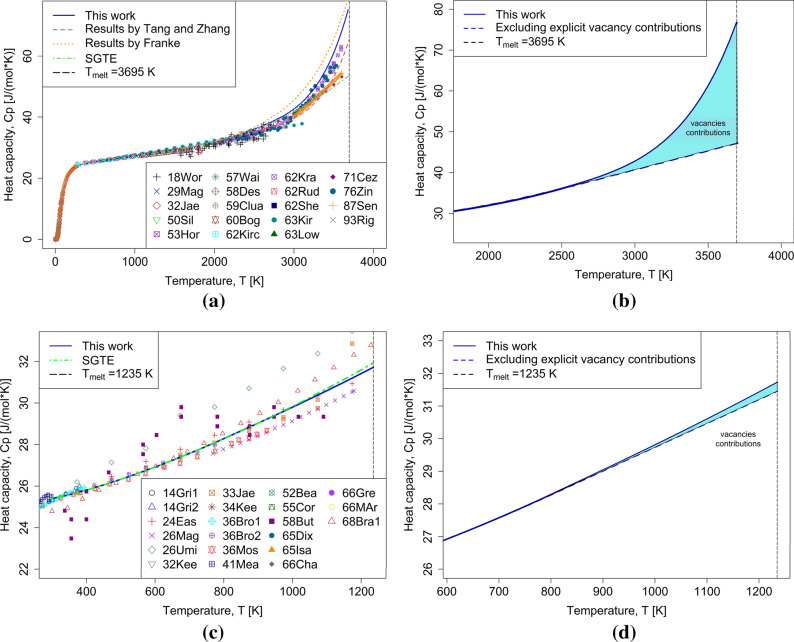
Proposed model heat capacity results for (**a**) BCC-W compared to results from Tang and Zhang^21^, Franke^20^ and SGTE^44^ and experimental data (**b**) vacancy contribution added by proposed model for BCC-W (**c**) FCC-Ag compared to results from SGTE44 and experimental data (**d**) vacancy contribution added by proposed model for FCC-Ag.

Having the highest melting point of all metals, BCC-W is considered a crucial case study for thermal vacancies. It represents the perfect example of how thermal vacancies can affect thermo-physical properties, such as heat capacity, in the vicinity of its melting point. For that reason, this element was investigated by Tang et al.^21^, who used the value, ${G}_{m}^{Va}=0.2 RT$, which was originally suggested by Franke^20^, who also chose to investigate the same BCC-W unary system in his work. It seemed ideal in this case to start with testing the proposed method on BCC-W and then compare the validity of the results accordingly. As shown in Fig. 6, the proposed model provided a very good description when compared with available experimental data for BCC-W and FCC-Ag. Furthermore, additional assessments were carried out for BCC-Ti, FCC-Al and FCC-Cu, and FCC-Ni, which are provided in the supplemental materials in Figs. S2–S5. Where available, the heat capacities of other descriptions including vacancies are included.

### Vacancy fraction as a function of temperature

**Figure 7:**
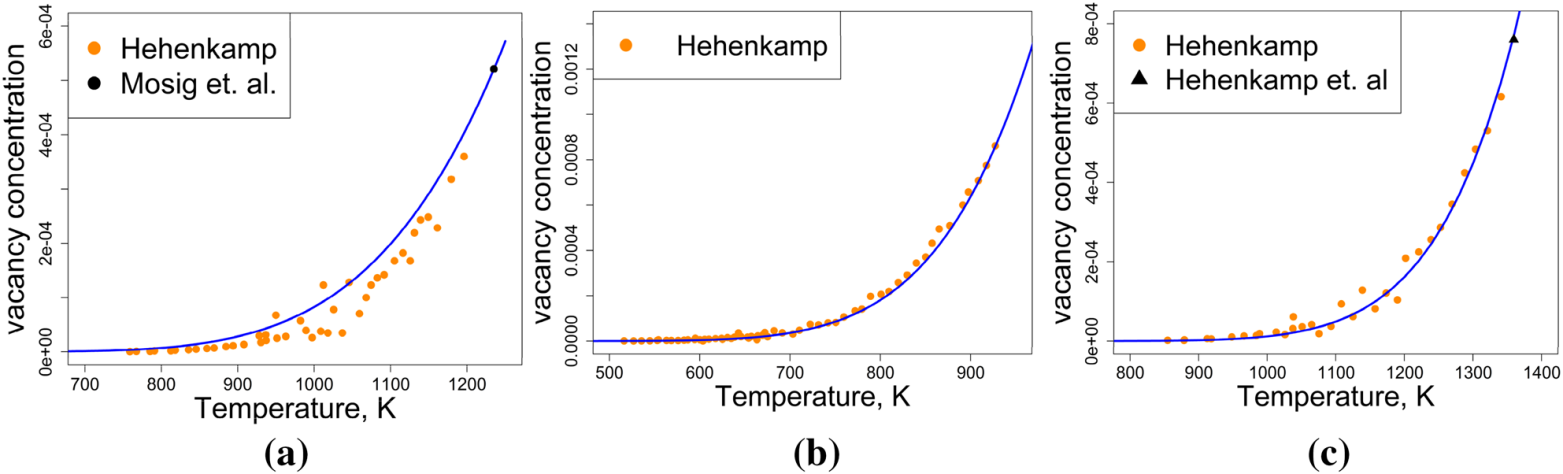
Vacancy concentration results from the proposed model for (**a**) FCC-Ag (**b**) FCC-Al (c) FCC-Cu compared with experimental data from Hehenkamp^36^, Hehenkamp et al.^52^, and Mosig et al.^53^.

 Experimental vacancy concentration measurements obtained using the DD method for FCC-Al, FCC-Ag, and FCC-Cu are compared against the results produced by the proposed model in Fig. 7. The vacancy concentration at the melting point was used during the parametrization, so perfect agreement at this temperature is not a surprise. However, the model also matches the experimental data not used during parametrization accurately, especially close to ${T}_{melt}$, where relative experimental errors become smaller due to the larger magnitude of vacancy concentrations, making measurements easier. This provides further support for the model’s validity and physical integrity.

### Gibbs energy and entropy of vacancy formation

**Figure 8:**
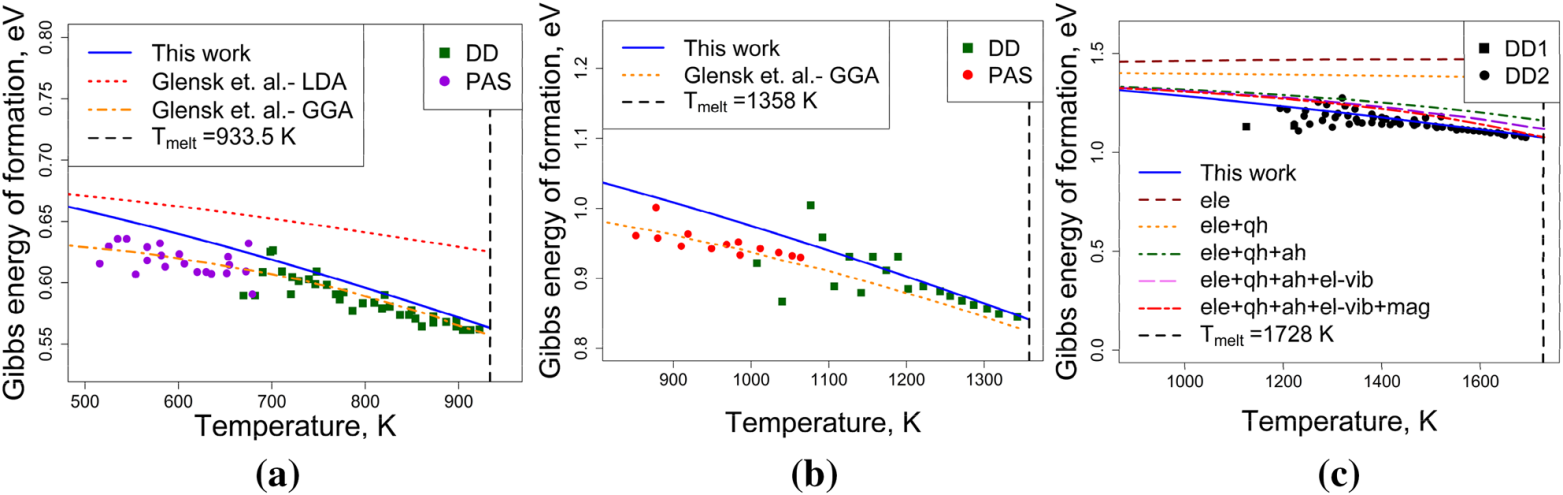
Gibbs energy of vacancy formation for (**a**) FCC-Al, (**b**) FCC-Cu, and (**c**) FCC-Ni, comparing the model performance against experimental data [PAS: positron annihilation spectroscopy^36^, DD: differential dilatometry^36,54–58^] and atomistic simulations^38^.

The vacancy concentration data from Fig. 7 can be converted to Gibbs energy of vacancy formation, which was used by Glensk et al.^38^ to benchmark their atomistic simulations against the experiments. Figure 8 compares this work against the data by Glensk et al. for FCC-Al and FCC-Cu and Gong et al.^31^ for FCC-Ni. It can be seen that the model formulated here reproduces the breakdown of the Arrhenius description^30^.

**Figure 9:**
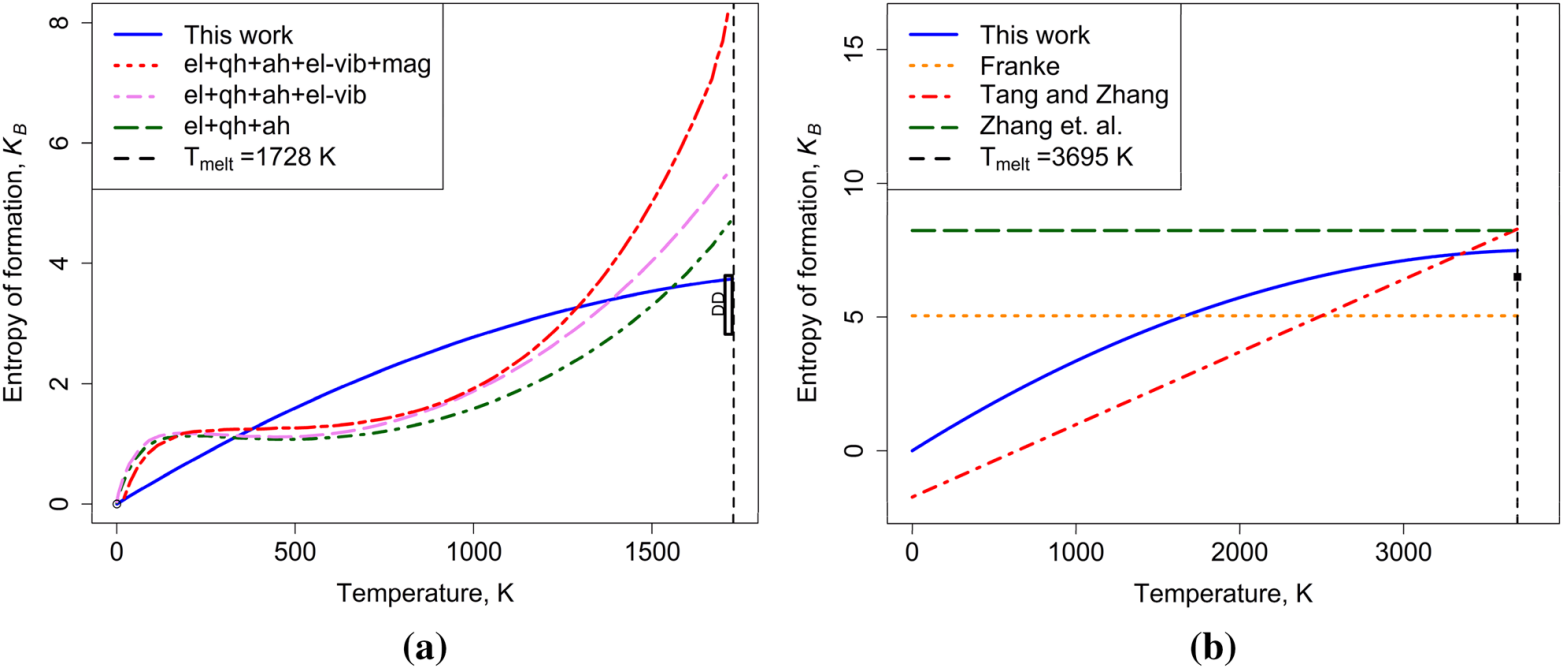
Entropy of vacancy formation for Ni and W, comparing the model performance for (**a**) FCC-Ni against experimental data^57^ and atomistic simulations^31^ (**b**) BCC-W against experimental data^6^ and Calphad models of Franke^20^, Tang and Zhang^21^, and Zhang^17^.

Entropy of vacancy formation is shown in Fig. 9 for FCC-Ni and BCC-W. For Ni, the model reproduces the trend obtained from atomistic simulations^31^ qualitatively, by reproducing a positive temperature dependence of entropy of vacancy formation at low temperatures and a reasonable order of magnitude at the melting point. For W, temperature-dependent entropy of vacancy formation is not available. However, the comparison to existing Calphad models^20,21^ clearly shows the different approach suggested here: The unphysical assignment of negative entropy of vacancy formation is avoided in the model suggested here.

### High-temperature extrapolation

All remaining unary systems were assessed using the proposed model. The results show that the proposed model was capable of maintaining sensible phase stability in all cases and can be found in supplementary materials, Figs. S6–S11.

### Avoiding Neumann–Kopp artifacts in solid solutions

The model’s heat capacity description managed to avoid Neumann–Kopp artifacts in solid solutions when compared with the existing SGTE description. Heat capacity for Cu-20% Zn is shown in Fig. S12.

### Binary and higher-order systems extrapolation

The FCC phase in the Cu–Zn system was extended to the model proposed here while keeping all Cu–Zn interactions the same. The resulting phase diagram does not change significantly, as shown in Fig. S13.

## Methods

The estimation of $\Delta {H}^{e}\left(0{\text{K}}\right)$ and ${V}_{m}$ was carried out using multiple ab initio calculations for supercell structures containing 15, 31, and 47 atoms for the FCC metals and 15, 23, 35 and 53 atoms for BCC-W, respectively, plus one vacancy. The calculations were performed through the VASP software using GGA pseudo-potentials. Volume and atomic positions have been relaxed. Thus, $\Delta {H}_{mix}\left(0{\text{K}}\right)$ was calculated for each investigated unary system (FCC-Al, FCC-Ag, FCC-Ni, BCC-W, FCC-Cu, FCC-Zn). From the same calculations, ${V}_{m}$ was obtained accordingly.

## Conclusion

The proposed thermochemical model joins the most recent experimental vacancy formation findings with the latest state-of-the-art physical understanding of vacancies and their effects. It is shown that the long-standing debate in the Calphad community on the choice of ${G}_{Va}$ can be settled since both options (${G}_{Va}$= 0 and ${G}_{Va}$= positive but small) can result in equivalent descriptions if parametrized using the methodology proposed here. Other Calphad-related concerns, such as extrapolation to multicomponent systems and re-stabilization of solids at high temperatures, were addressed successfully in this work. Additionally, the model results shows excellent agreement with experimental heat capacity and vacancy concentration data as well as theoretical entropy of vacancy formation data. The proposed model can easily be applied to other elements or compounds if experimental and ab initio data are available. Explicitly accounting for thermal vacancies contributions in multicomponent solutions is thus possible and can be very beneficial when modelling processes like diffusion or precipitation.

## Supplementary Information

Supplementary Information.

## Data Availability

The data that support the findings of this study are available from the corresponding author upon reasonable request.
