# Supplementary material for: Including state-of-the-art physical understanding of thermal vacancies in Calphad models
Source: Sci Rep. 2022 Aug 4;12:13385. doi: 10.1038/s41598-022-16926-5 (PMC9352709; doi:10.1038/s41598-022-16926-5)
Supplement: Supplementary file 1 — Supplementary Information. [file 41598_2022_16926_MOESM1_ESM.docx]

Supplementary materials for the “Including state-of-the-art physical understanding of thermal vacancies in Calphad models” article. Submitted by A. Obaied, I. Roslyakova, and M. to Baben

1. Supplementary Text
   1. Choice of $G_{Va}$

As introduced in the main body of the text, the choice of $G_{Va}$ has been under debate in the Calphad community for a long time. While the Calphad modelling software used here, FactSage, enables the setting of maximum values for constituents mole fractions, other software packages are not able to do this which has led to the necessity to use $G_{Va}$ > 0. Fig. S1 compares Gibbs energy of Ag at the melting point for three different values of $G_{Va}$. $G_{Va}=10\frac{J}{K mol}T$ can be considered the upper boundary above which significant changes to the solutions Gibbs energy at 20% vacancy concentration can be observed. Therefore, $G_{Va}=0$ together with $y_{Va}$ < 0.2 and $G_{Va}=2\frac{J}{K mol}T$ are used throughout this work, leading to equivalent results.

- 1. High-temperature extrapolation

Several thermodynamic assessments argued the necessity of setting the value of $G_{Va}$ higher than zero as the only way of stabilizing the phase equilibria. It was shown in Figure 4 FCC-Al that the proposed model including the limit of $y_{Va}<0.2$ managed to solve this problem without the need to compromise its physical integrity. The heat capacity and Gibbs energy description for rest of the assessed elements (FCC-Ag, FCC-Cu, FCC-Ni and FCC-Zn, and BCC-W) can be seen Fig. S2 to S9. It is important to point out that for the case of FCC-Zn, no equilibrium vacancy concentration can be found in literature as Zn crystallizes in HCP structure. For this reason, the recommended value for $a$ and $b$ for FCC-Zn were assumed to be the average value of FCC-Ni, FCC-Ag, FCC-Al, and FCC-Cu, respectively. For all elements, Gibbs energy extrapolates reasonably up to at least 6000 K.

- 1. Avoiding Neumann-Kopp artifacts in solid solutions

In Fig S10 the calculated heat capacity of the FCC phase containing 30% Zn and 70% Cu is shown and compared with the results from the SGTE database. The SGTE description clearly contain Neumann-Kopp artifact, i.e. a discontinuity at the melting temperature of Cu, which is inherited from the pure element. The proposed model description avoids these artifacts which is in itself an improvement. Experimental data to corroborate the absolute difference in heat capacity observed is unfortunately missing.

- 1. Binary and higher-order systems extrapolation

The SGTE thermodynamic Cu-Zn assessment was modified to adapt the new proposed model and the resulting phase diagram can be seen as an overlay on the original phase diagram from the SGTE database in Fig. S11. It is evident that no instabilities affect the phase equilibria due to the use of our proposed model and the equilibria are also nearly unchanged. This indicates that it is possible to implement this model on large scale in already existing Calphad databases without the need to extensively re-model all binary systems.

1. Supplementary Figures
   1. Choice of G_Va_

|  |
| --- |
| **Fig. S1** Gibbs energy of 1 mol Ag at the melting point (1335 K) as function of vacancy concentration using $G_{Va}$ = 0, $G_{Va}=2\frac{J}{K mol}T$ and $G_{Va}=10\frac{J}{K mol}T$. It is seen that in the limit of applicability ($c_{Va}^{eqm}<0.2)$, all three assumptions lead to equivalent results. |

- 1. Heat capacity and Gibbs energy figures for assessed FCC-Al, FCC-Cu, FCC-Zn, and FCC-Ni unary systems

| 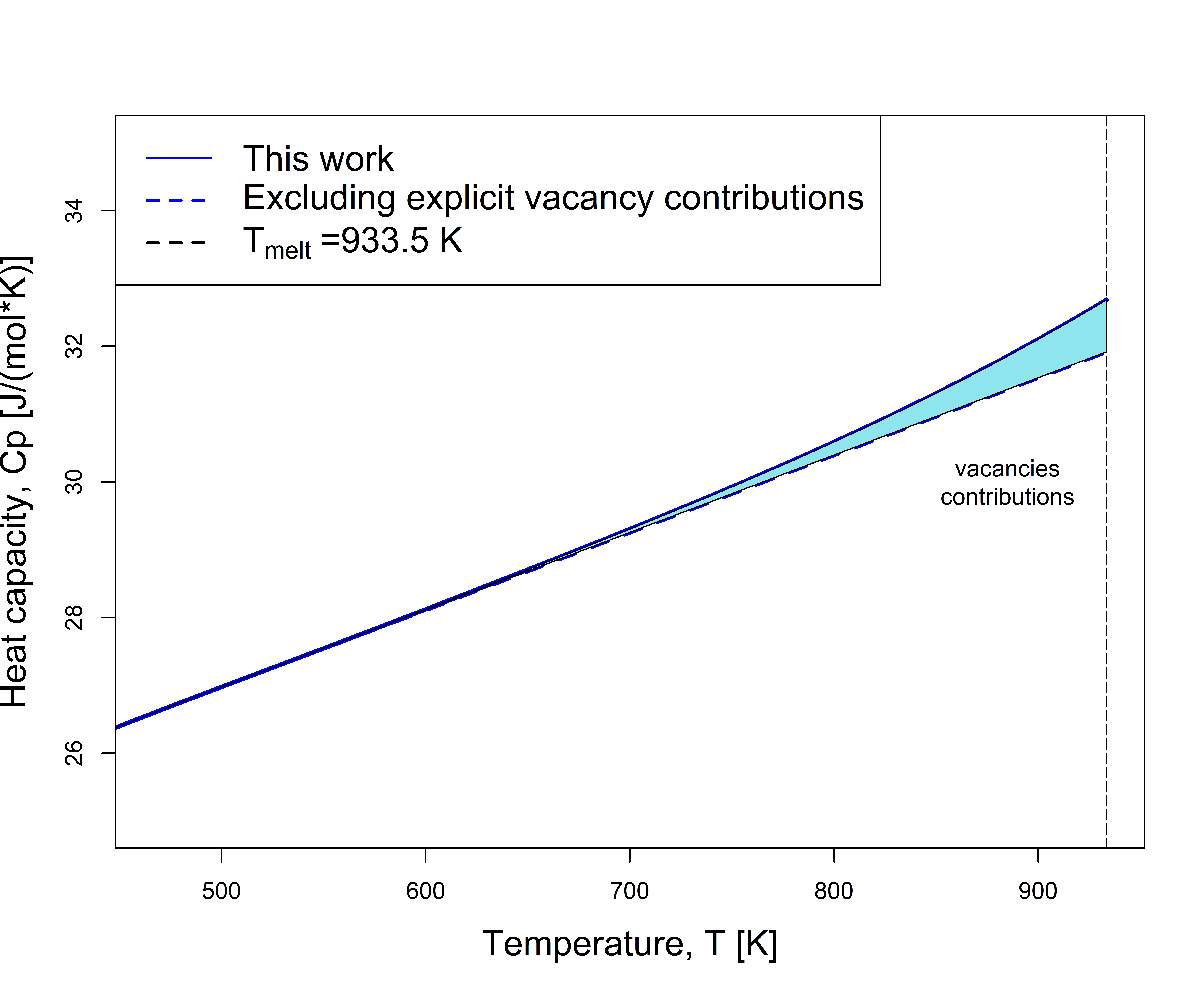 |
| --- |
| **Fig.** **S2** Heat capacity description of FCC-Al plotted using the proposed model with comparison to results from Grabowski^6^ SGTE^7^ |

| 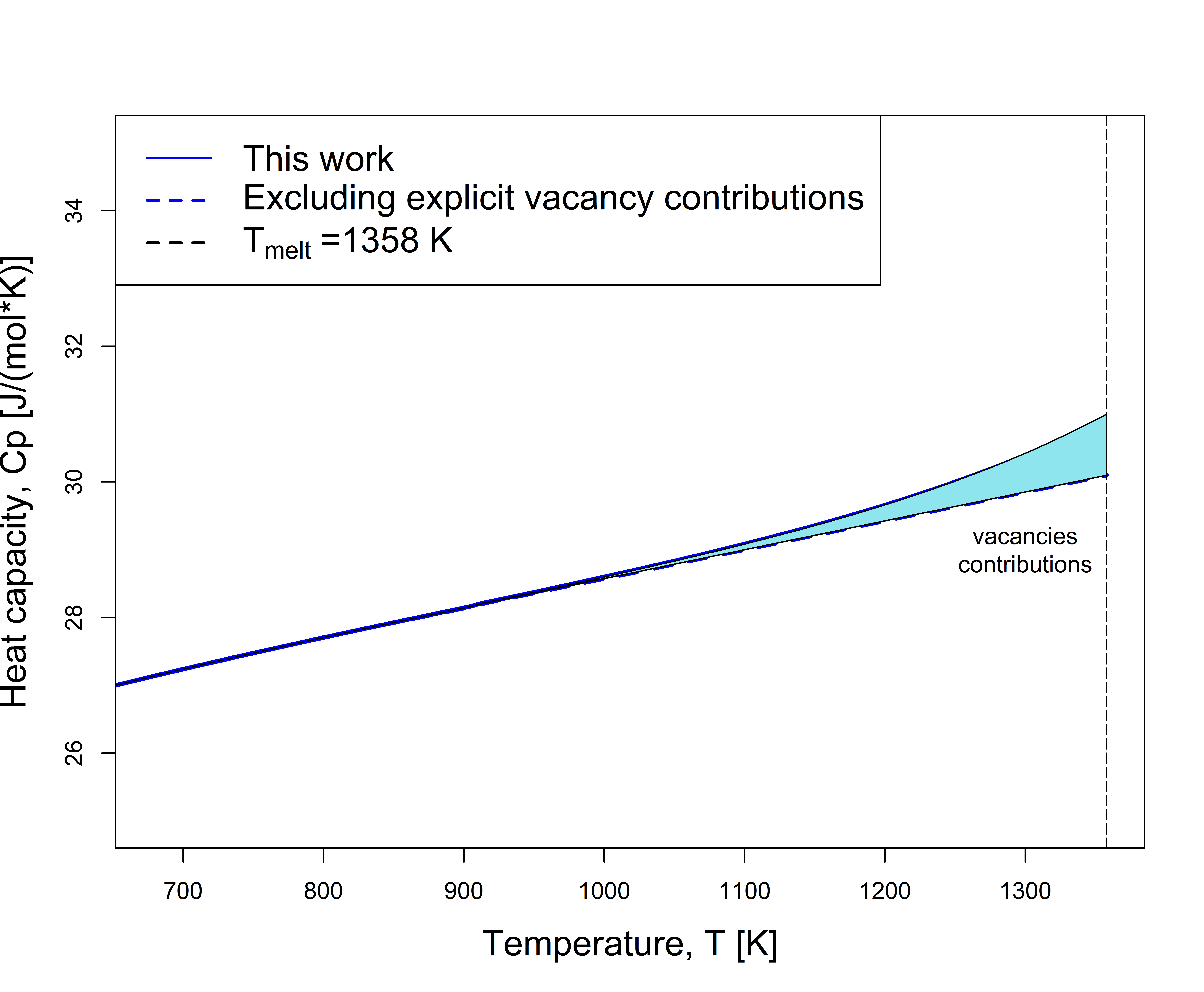 |
| --- |
| **Fig.** **S3** Heat capacity description of FCC-Cu plotted using the proposed model with comparison to results from SGTE^7^ |

| 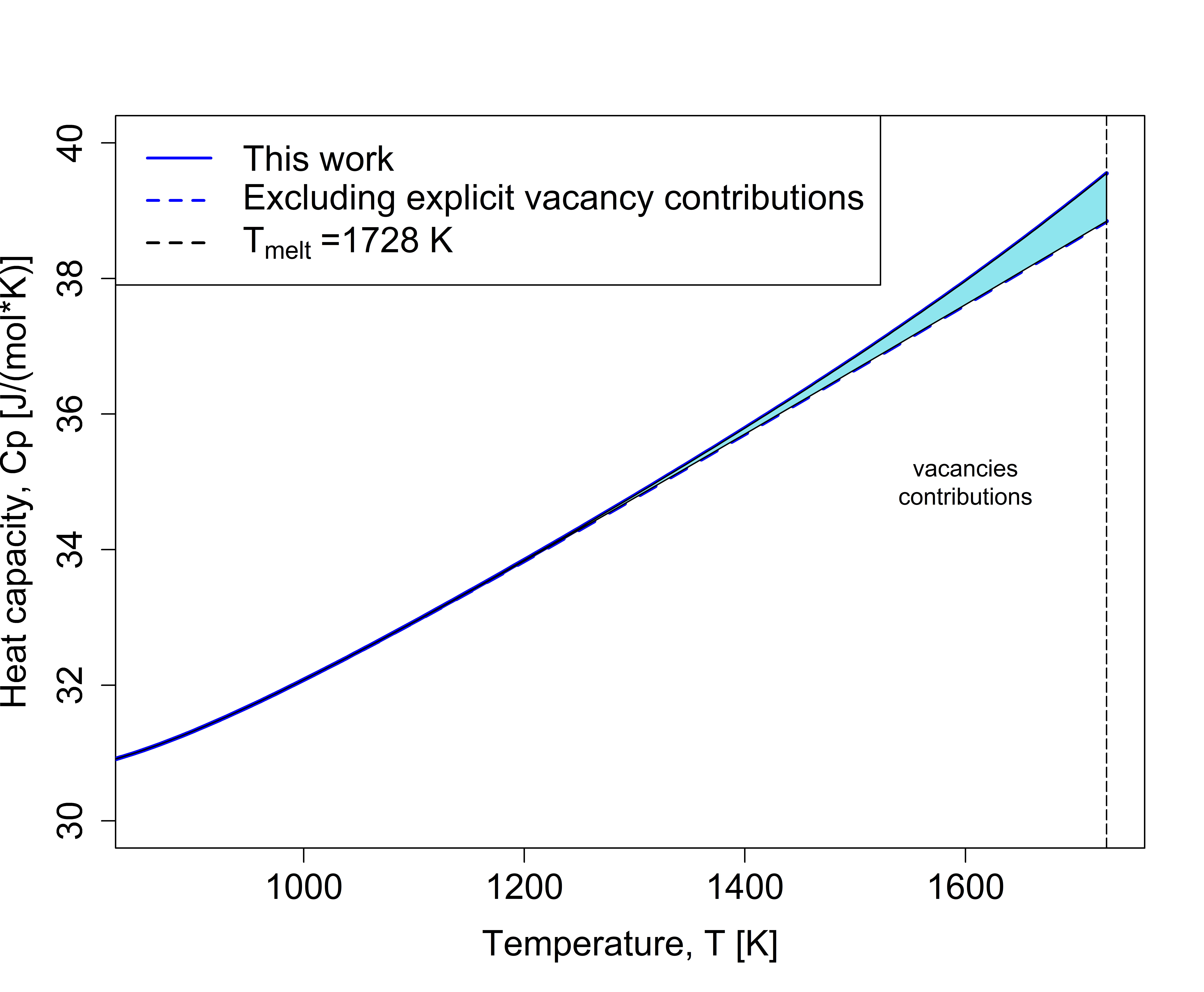 |
| --- |
| **Fig. S4** Heat capacity description of FCC-Ni plotted using the proposed model with comparison to results from SGTE^7^ |

| 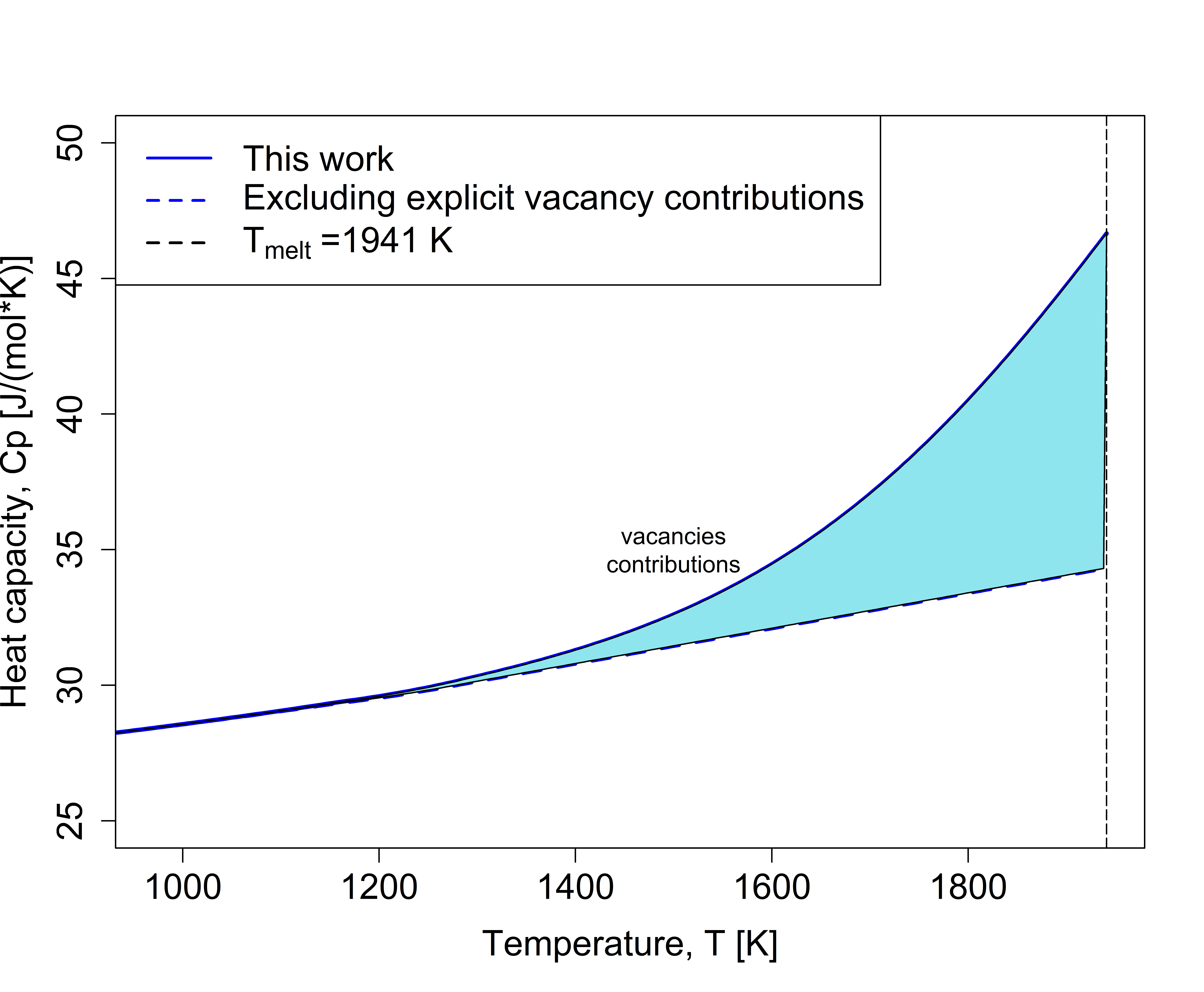 |
| --- |
| **Fig. S5** Heat capacity description of BCC-Ti plotted using the proposed model with comparison to results from SGTE^7^ |

| 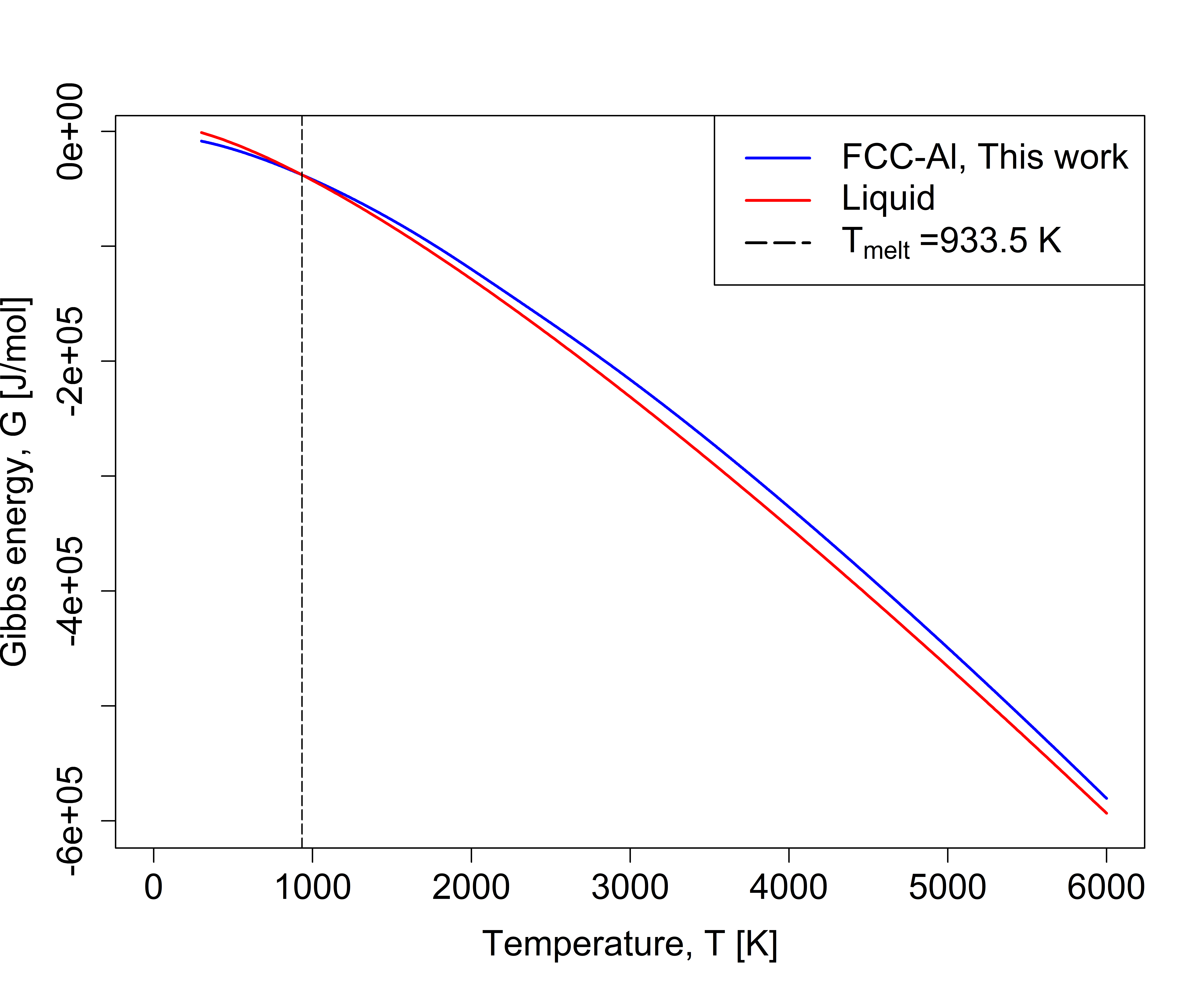 |
| --- |
| **Fig. S6** Gibbs energy description FCC-Al |

| **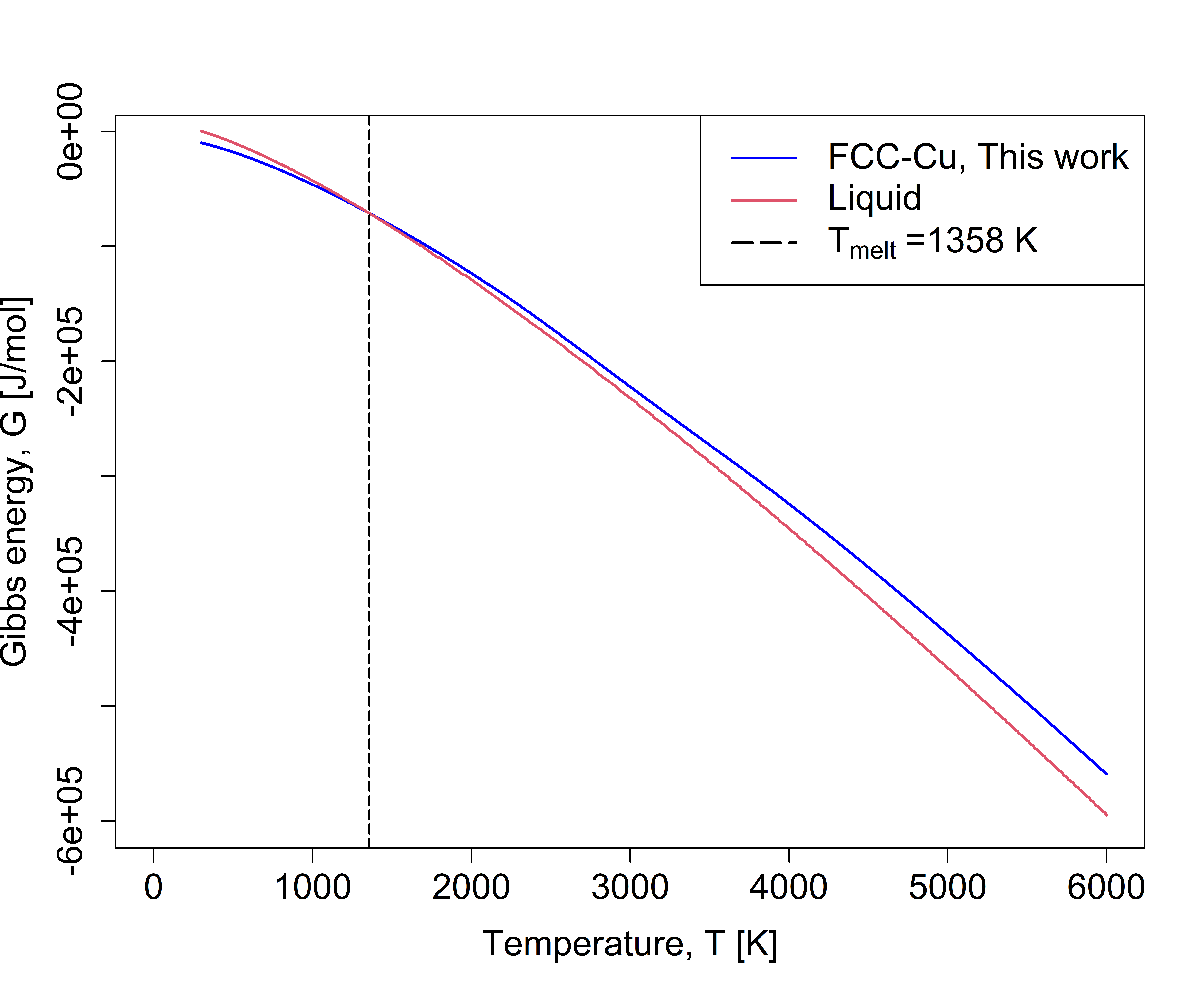** |
| --- |
| **Fig. S7** Gibbs energy description FCC-Cu |

| 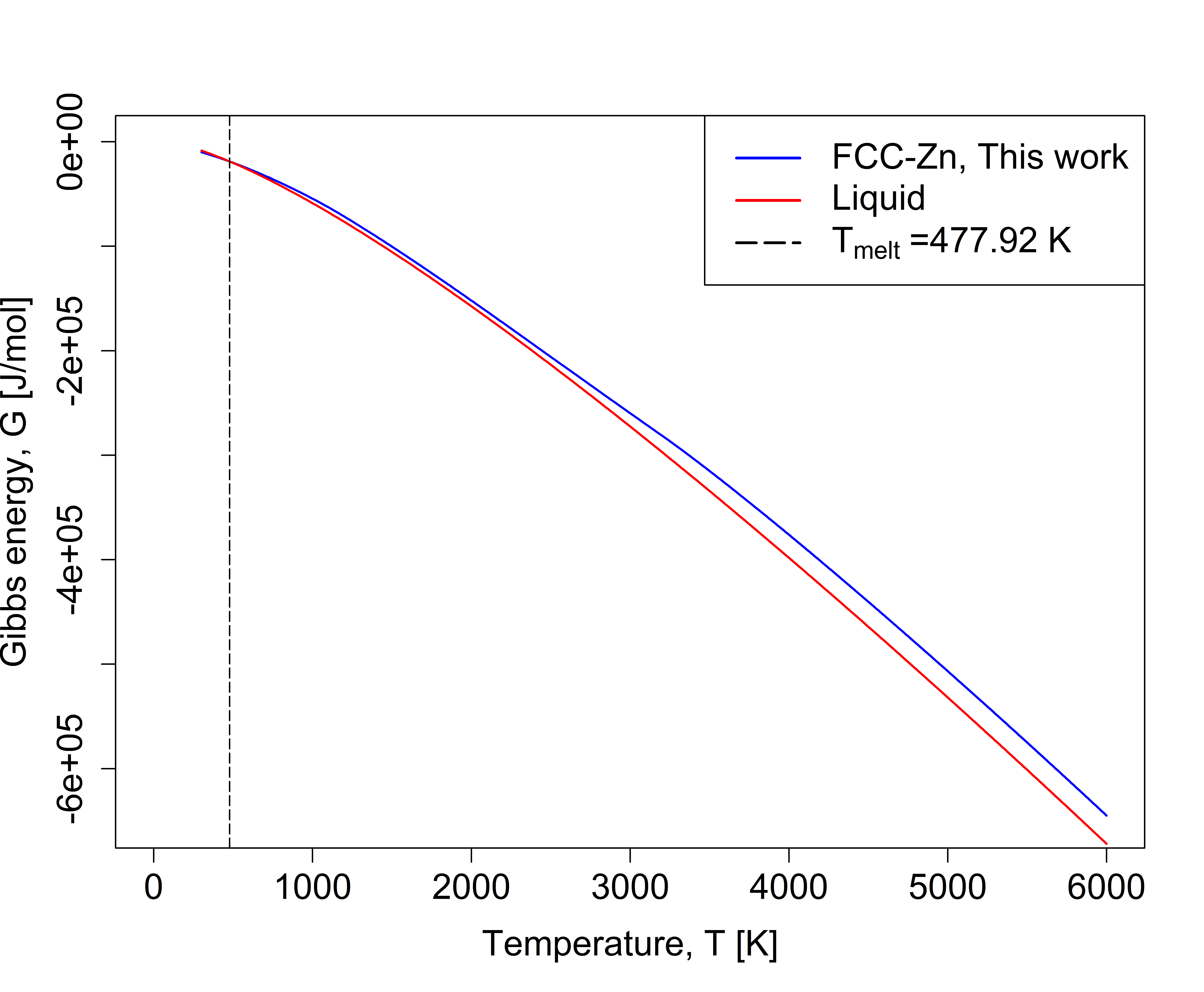 |
| --- |
| **Fig. S8** Gibbs energy description FCC-Zn |

| **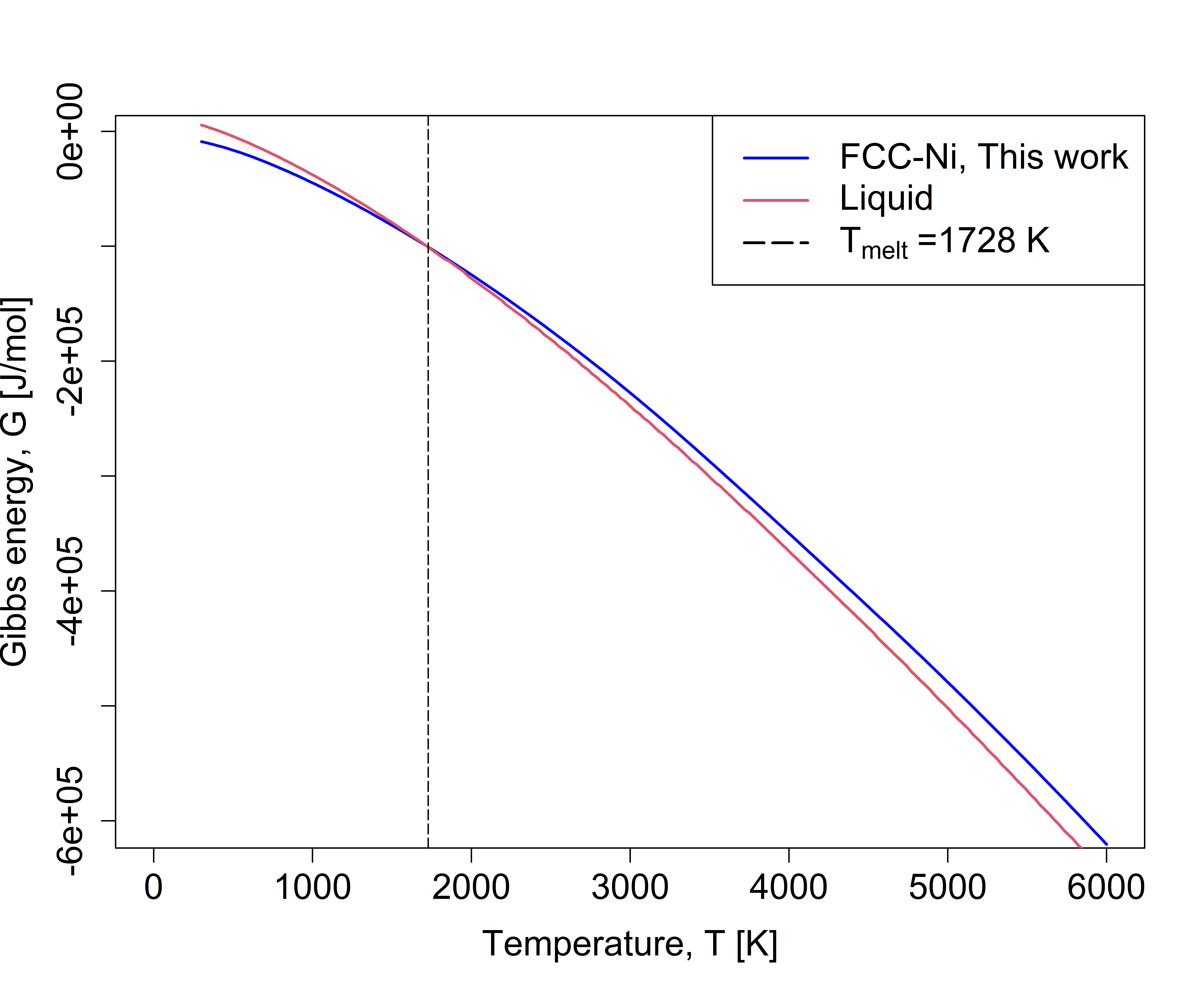** |
| --- |
| **Fig. S9** Gibbs energy description FCC-Ni |

| 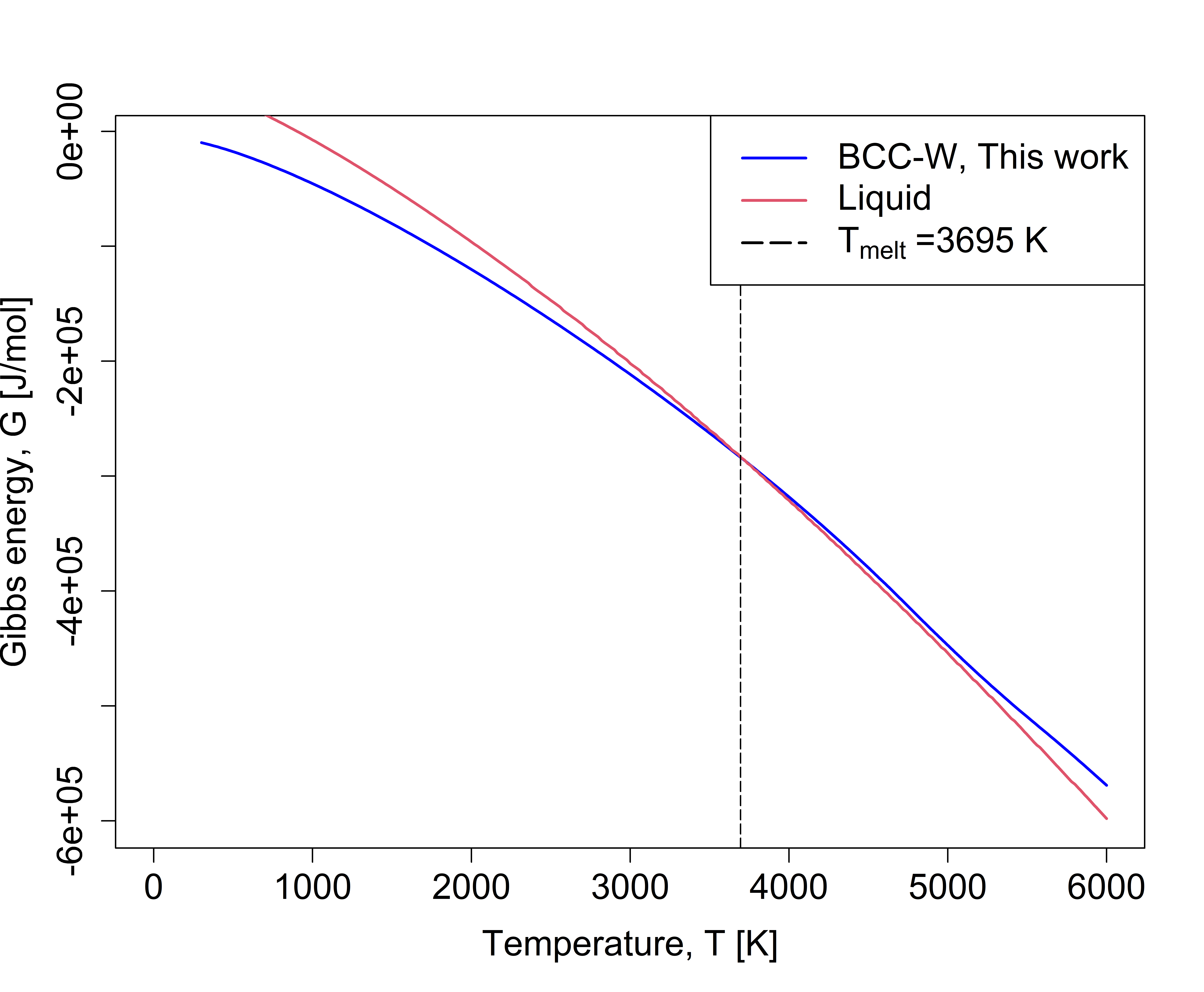 |
| --- |
| **Fig. S10** Gibbs energy description BCC-W |

| 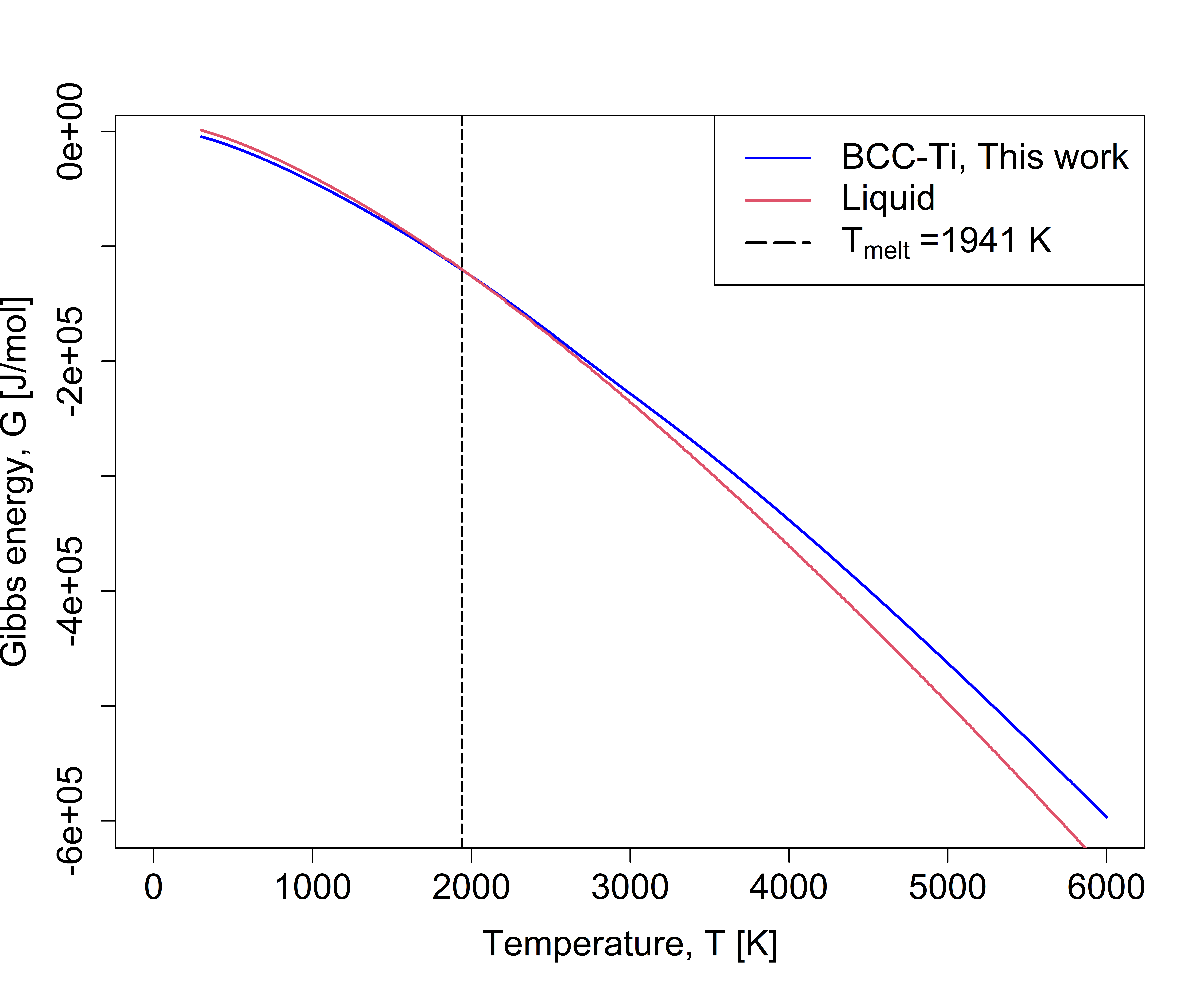 |
| --- |
| **Fig. 11** Gibbs energy description BCC-Ti |

- 1. Avoiding Neumann-Kopp artifacts in solid solutions

|  |
| --- |
| **Fig.** **S12** Binary heat capacity description for 30% Zn and 70% Cu in the FCC phase |

- 1. Binary and higher-order systems extrapolation

| 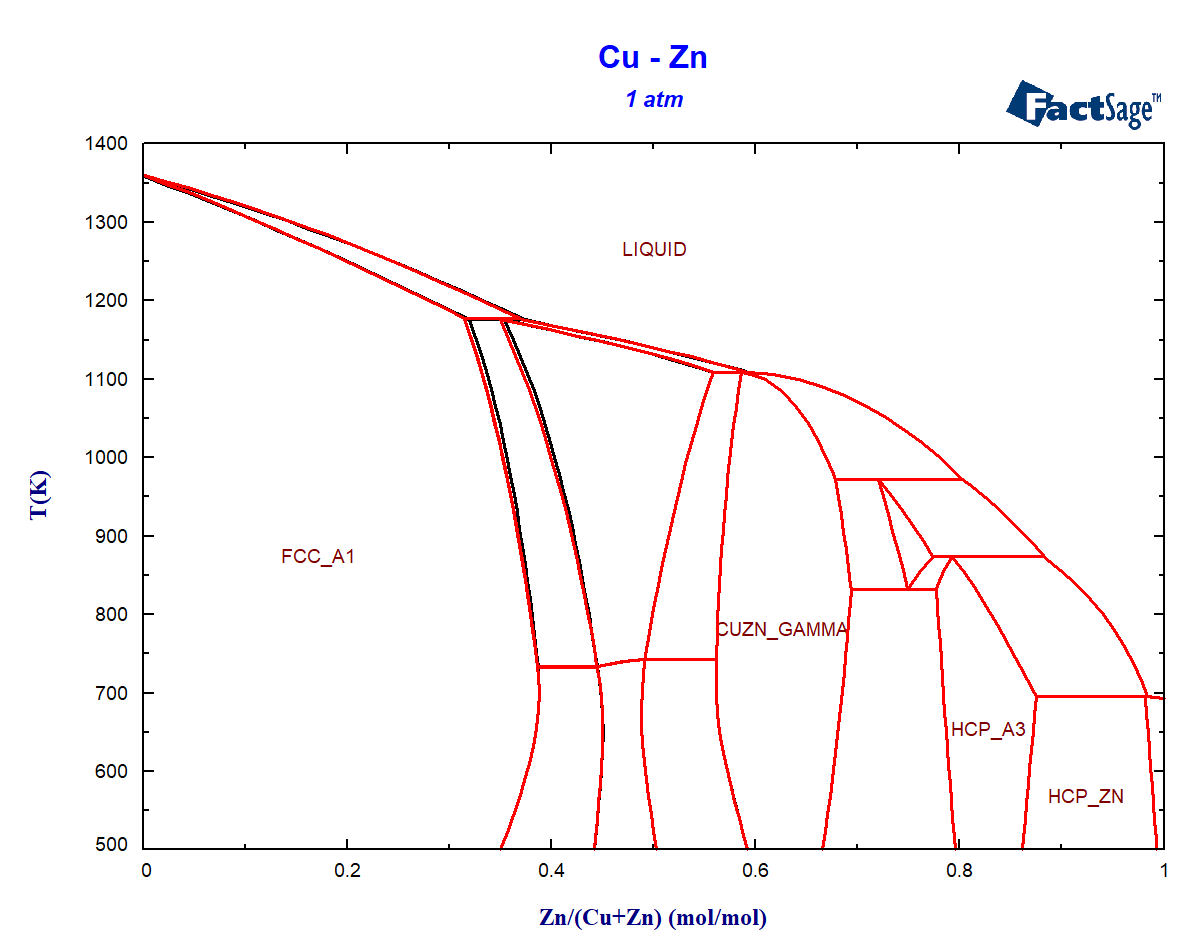 |
| --- |
| **Fig.** **S13** Cu-Zn phase diagram. Red: This work. Black: SGTE |

1. Supplementary tables

| Element | $\Delta H^{e}\left( 0K \right)$(eV)  This work | $\Delta H^{e}$(eV)  From Literature |
| --- | --- | --- |
| FCC-Al | 0.70 | 0.66 |
| FCC-Ag | 0.82 | 1.06 |
| BCC-W | 3.65 | 3.30 |
| FCC-Cu | 1.17 | 1.05 |
| **Table S1:** Comparing calculated values of $\Delta H^{e}\left( 0K \right)$ with values from literature for W^8^, Al^9^, Ag^10^, and Cu^11^ | | |

| Element | Interaction parameters | α |
| --- | --- | --- |
| FCC-Al | $L_{E}=68231-0.0239 T^{2} +8.611\times{10}^{-6} T^{3}$  $L_{V}=0.0075$ | 50% |
| FCC-Ag | $L_{E}=79241-0.0015 T^{2} +4.049\times{10}^{-7} T^{3}$  $L_{V}=0.0075$ | 50% |
| FCC-Cu | $L_{E}=112967 -0.0258 T^{2}+6.335\times{10}^{-6} T^{3}$  $L_{V}=0.0048$ | 50% |
| FCC-Zn | $L_{E}=37704 -0.0171 T^{2}+5.117\times{10}^{-6} T^{3}$  $L_{V}=0.0393$ | 31% |
| FCC-Ni | $L_{E}=136549 -0.0165 T^{2} +3.181\times{10}^{-6} T^{3}$  $L_{V}=0.0068$ | 50% |
| BCC-W | $L_{E}=352437 -0.0338 T^{2} +4.573\times{10}^{-6} T^{3}$  $L_{V}=0.0064$ | 75% |
| BCC-Ti | $L_{E}=151323.33 -0.0441 T^{2} +1.136\times{10}^{-5} T^{3}$ | 75% |
| **Table S2:** Interaction parameters for all assessed unary systems | | |

**References**

1. Schick, M., Watson, A., to Baben, M. & Hack, K. A Modified Neumann–Kopp Treatment of the Heat Capacity of Stoichiometric Phases for Use in Computational Thermodynamics. *J. Phase Equilibria Diffus.* **40**, 104–114 (2019).

2. Saenko, I., Engelhardt, H., Hornig, P., Fabrichnaya, O. & Lippmann, S. Specific heat capacity of the intermetallics ε-Cu3Sn, η/η′-Cu6Sn5, Al3Ni and Al3Ni2. *Calphad* **74**, 102294 (2021).

3. Rank, M., Gotcu, P., Franke, P. & Seifert, H. J. Thermodynamic investigations in the Al-Fe system: Heat capacity measurements of three intermetallic phases. *Intermetallics* **94**, 73–82 (2018).

4. Abe, T., Hashimoto, K. & Shimono, M. Description of Thermal Vacancies in the CALPHAD Method. *Mater. Trans.* **59**, 580–584 (2018).

5. Abe, T., Shimono, M., Hashimoto, K. & Kocer, C. A description of vacancy complexes in an FCC solid solution within the framework of the CALPHAD Method. *Calphad* **63**, 100–106 (2018).

6. Grabowski, B., Ismer, L., Hickel, T. & Neugebauer, J. Ab initio up to the melting point: Anharmonicity and vacancies in aluminum. *Phys. Rev. B - Condens. Matter Mater. Phys.* **79**, 134106 (2009).

7. Dinsdale, A. SGTE data for pure elements. *Calphad* **15**, 317–425 (1991).

8. Chekhovskoi, V. Y. Energy generation of vacancies and their concentration i n tungsten. *Metallofizika* **3**, 116–119 (1981).

9. Shukla, R. C., Plint, C. A. & Ditmars, D. A. Aluminum. II. Derivation of C v0from C p and comparison to C v0 calculated from anharmonic models. *Int. J. Thermophys. 1985 65* **6**, 517–532 (1985).

10. Ascoli, A., Guarini, G. & Queirolo, G. T. Equilibrium vacancy resistivity in pure noble metals. *CRYST LATTICE DEFECTS* **1**, 159–163 (1970).

11. Solov’ev, V. A. Formation of local melting regions in a solid body near the melting temperature. *Russ. Metall. 2013 20135* **2013**, 375–380 (2013).
